# Precise Synthesis, Properties, and Structures of Cyclic Poly(*ε*-caprolactone)s

**DOI:** 10.3390/polym10060577

**Published:** 2018-05-23

**Authors:** Li Xiang, Wonyeong Ryu, Heesoo Kim, Moonhor Ree

**Affiliations:** 1 Department of Chemistry, Division of Advanced Materials Science, and Polymer Research Institute, Pohang University of Science and Technology, Pohang 37673, Korea; lea1990@postech.ac.kr (L.X.); rwy0511@postech.ac.kr (W.R.); 2 Department of Microbiology and Dongguk Medical Institute, Dongguk University College of Medicine, Gyeongju 38066, Korea

**Keywords:** cyclic poly(*ε*-caprolactone), linear poly(*ε*-caprolactone), precise synthesis, thermal properties, hydrolytic degradation, crystallization, morphological structure

## Abstract

Cyclic PCL (*c*-PCL) has drawn great attention from academia and industry because of its unique, unusual structure and property characteristics due to the absence of end groups in addition to the biocompatibility and biodegradability of its linear analogue. As a result of much research effort, several synthetic methods have been developed to produce *c*-PCLs so far. Their chain, morphology and property characteristics were investigated even though carried out on a very limited basis. This feature article reviews the research progress made in the synthesis, morphology, and properties of *c*-PCL; all results and their pros and cons are discussed in terms of purity and molecular weight distribution in addition to the cyclic topology effect. In addition, we attempted to synthesize a series of *c*-PCL products of high purity by using intramolecular azido-alkynyl click cyclization chemistry and subsequent precise and controlled separation and purification; and their thermal degradation and phase transitions were investigated in terms of the cyclic topology effect.

## 1. Introduction

Linear poly(*ε*-caprolactone) (PCL) has attracted great attention from both academia and industry because of its wide application potential ranging from surgical sutures, stents, prosthetics, artificial implants, wound dressings, tissue engineering, drug delivery systems, to environmentally friendly material systems, based on its biocompatibility and biodegradability [1,2,3,4,5].

The research interest has recently been extended to topological PCLs. In particular, the cyclic topology, namely cyclic PCL (*c*-PCL) has drawn attraction because of its unique properties due to the absence of end groups [6,7,8,9,10,11,12]. The unusual properties render *c*-PCL more versatile in various fields. A research effort was first made to develop synthetic methods of *c*-PCL. As a result, several synthetic schemes were reported [6,7,8,9,10,11,12,13,14,15,16,17,18,19,20,21,22,23,24,25,26,27,28,29,30]. The synthesized products were examined in terms of properties and structures [6,7,8,10,16,17,18,20,27,28,31,32,33,34,35]. Nevertheless, *c*-PCL synthesis is still confronted by critical issues: unreacted linear PCL precursor residues and their removal, byproducts and their removal, low reaction yields, long reaction times, limited ring sizes, and so on. Additionally, the properties and structures of *c*-PCL products have been investigated on a very limited basis.

In this feature article, we review the synthetic schemes, properties, and morphological structures of *c*-PCL reported in the literature so far. In addition, we discuss new results of the precise synthesis and thermal properties of *c*-PCL recently achieved in our laboratory. In our study, a series of *c*-PCL products was synthesized by using the intramolecular click chemistry of linear PCL precursors possessing an azido group on one chain end and an alkynyl group on the other chain end; their thermal properties were examined by using thermogravimetry (TGA) and differential scanning calorimetry (DSC).

## 2. Synthesis

Much research effort has been made to synthesize *c*-PCLs. As a result, several synthetic methods have been developed so far, and can be classified into three major families: (i) ring-expansion polymerizations; (ii) intermolecular cyclizations; (iii) intramolecular cyclizations. In this section, the synthetic schemes reported in the literatures are reviewed and their pros and cons are discussed. The purity of *c*-PCL is critical in understanding its structure, properties and the development of its applications. Thus, its workup process is discussed in detail from the view of purity. At the end of this section, our precise synthesis works are additionally introduced together with purification processes, providing highly pure *c*-PCL products with low polydispersity indices (PDIs).

### 2.1. Ring-Expansion Polymerization

**Scheme 1:**
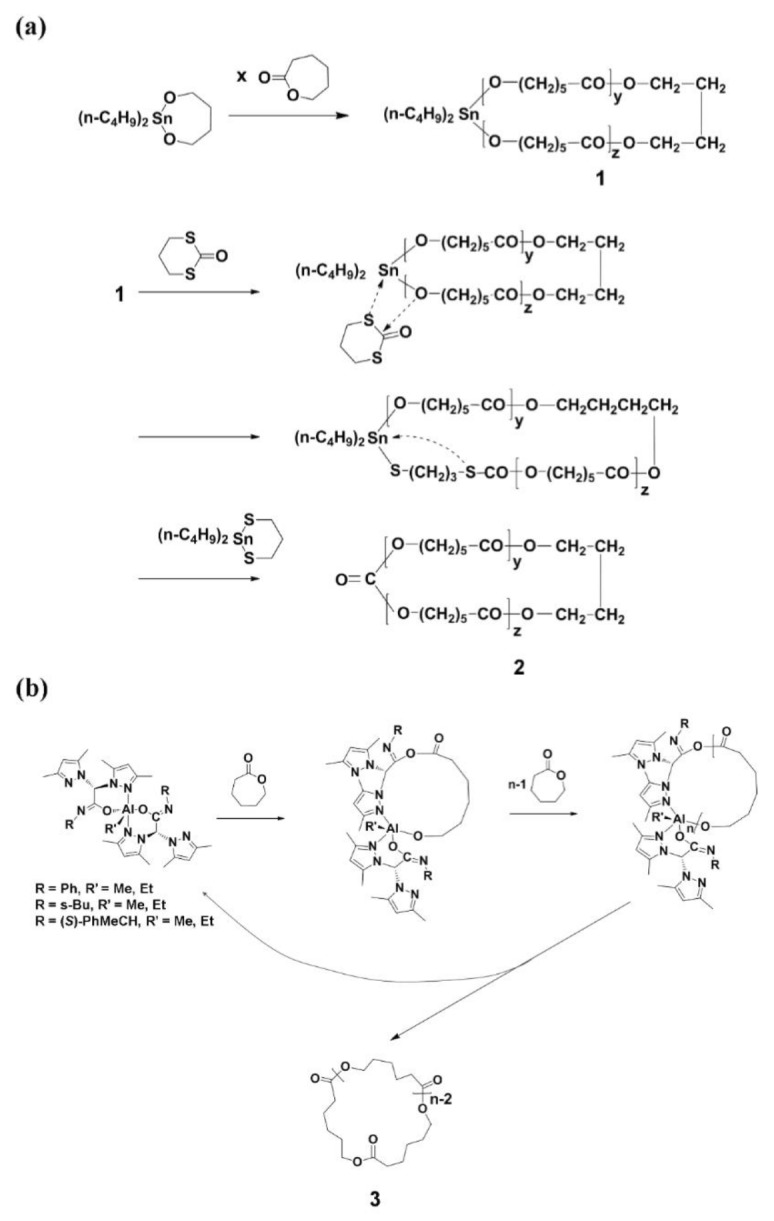
Ring-expansion polymerization (REP) of *ε*-caprolactone (CL): (**a**) 2,2-dibutyl-2-stanna-1,3-dioxebutane [*n*-Bu_2_Sn(OCH_2_CH_2_CH_2_CH_2_O)] was used as a cyclic initiator [13,14,15,16,17]; (**b**) alkyl-organoaluminum initiators were used as initiators [18].

A ring-expansion polymerization (REP) of *ε*-caprolactone (CL) was first succeeded in 1998 by using 2,2-dibutyl-2-stanna-1,3-dioxebutane [*n*-Bu_2_Sn(OCH_2_CH_2_CH_2_CH_2_O)], a cyclic initiator synthesized from di-*n*-butyltin dimethoxide [*n*-Bu_2_Sn(OMe)_2_] and 1,4-dihydroxybutane (Scheme 1a) [13,14,15,16,17]. The REP reaction was carried out at 60~180 °C in bulk. The degree of polymerization (DP) was proportional to the [monomer]/[initiator] (=[M]/[I]) up to 1000 (which corresponds to a number-average molecular weight $\bar{M_{n}}$ of 190,000 g/mol). The polydispersity index (PDI) ranged 1.42~1.71 and increased with the [M]/[I] ratio, reaction time and temperature. The reaction conversion was >90%. Overall, this REP is a good approach to synthesize *c*-PCL. However, this method was found to have three drawbacks. The first one is the formation of cyclic oligomers as byproducts via thermal back-biting degradations; the amount of such byproducts increased with reaction temperature and time; the lowest yield of byproducts was 1~2 wt %. The second one is the formation of *c*-PCL containing the 2,2-dibutyltin component in the ring structure; the presence of tin metal component may cause toxicity in some applications. The tin metal component could be eliminated via an insertion–elimination mechanism using 2-oxo-1,3-dithiane. However, the obtained *c*-PCL product behaves with a low stability due to the carboxylic anhydride unit formed in the ring structure. The final drawback is the formation of *c*-PCL with relatively high PDI values.

For the REP reaction of CL, monoalkyl-organoaluminums were introduced in 2013 as a new initiator system (Scheme 1b) [18]. Their cyclic initiator form would be generated through the nucleophilic attack upon CL by the scorpionate ligand followed by macrolactonization via the continuous insertion of CL monomers without the involvement of the alkyl (methyl or ethyl) group directly attached to the aluminum center. The REP reaction produced *c*-PCL via the intramolecular chain-transfer reaction and the regeneration of the monoalkyl-organoaluminum initiator. The REP reactions were conducted in toluene at 70~130 °C. The reaction yield ranges from 91 to 99%, depending on the alkyl substituent, [M]/[I] ratio, temperature, and time; the obtained *c*-PCL products were obtained with $\bar{M_{n}}$ = 18,690~52,020 g/mol and PDI = 1.02~1.37; both $\bar{M_{n}}$ and PDI were increased with reaction time. All results collectively informed that the monoalkyl-organoaluminum initiator is quite novel for the REP of CL and its performance is much better than that of 2,2-dibutyl-2-stanna-1,3-dioxebutane initiator. Moreover, it is quite unique that this synthetic method can produce *c*-PCLs without any chemical heterogeneities in the ring. However, there are some concerns as follows. First, the intramolecular chain-transfer reaction was proposed for the termination step. This chain transfer reaction may have a chance of causing side reactions, possibly producing byproducts including cyclic oligomers. Second, it is still necessary to verify the presence of an aluminum-containing component or parts of the initiator within the obtained *c*-PCL. Third, how can the metal-containing initiator be eliminated from the *c*-PCL after the REP reaction? Fourth, can *c*-PCL with $\bar{M_{n}}$ higher than 60,000 g/mol be achievable? Lastly, the PDI value is still large; can it be improved to a lower value?

### 2.2. Intermolecular Cyclization

**Scheme 2:**
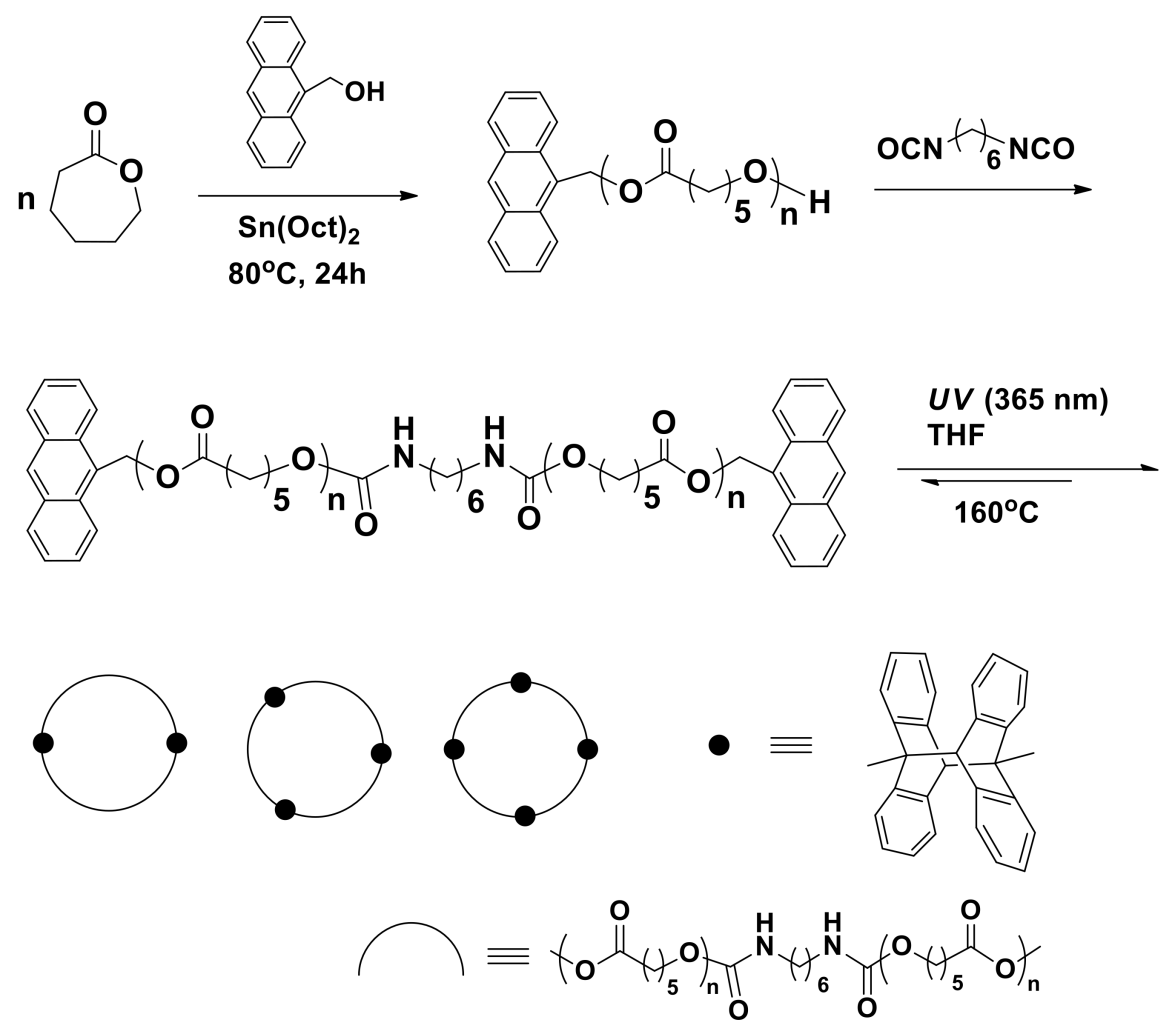
Ring-opening polymerization of CL using an aromatic alcohol as an initiator; the post modification and intermolecular cyclization of the linear PCL product [19].

The synthesis of *c*-PCL bearing cycloadded anthracene units was reported (Scheme 2) [19]. *α,ω*-Anthracene-terminated linear PCLs were synthesized at 80~110 °C in toluene under argon atmosphere by a one-pot reaction composed of two steps. In the first-step, mono-anthracene-terminated PCLs were synthesized from CL with the aid of 9-anthracenemethanol initiator and tin(II) 2-ethylhexanoate (Sn(Oct)_2_) catalyst; in the second-step, the coupling reaction of mono-anthracene-terminated PCLs was conducted by using 1,6-hexane diisocyanate. The obtained dianthracene-terminated PCLs were measured to have $\bar{M_{n}}$ = 3270~6780 g/mol and PDI = 1.38~1.99, depending on the [M]/[I] ratio, temperature, and time in the polymerization; the polymerization yields ranged between 72% and 98%. Dianthracene-terminated PCL underwent [4+4] a cyclization reaction in tetrahydrafuran (THF) over the polymer concentration range of 10 to 100 mg/mL by ultraviolet (UV) exposure (365 nm wavelength); the exposure time was in the range of 120~168 h. The cyclic PCL products were purified by precipitation in methanol and subsequent filtration and drying. The obtained product was a cyclic dimer to cyclic tetramer, depending on the linear precursor polymer concentration and UV-exposure time in the photocyclization reaction. The *c*-PCL products revealed PDI = 2.06~2.26, depending on the ring size; larger *c*-PCL showed higher PDI value. Overall, the photoinduced cyclization is a good approach to produce cyclic multimers. However, this method should clarify several concerns or questions as follows. First, *α*,*ω*-anthracene-terminated linear PCL precursors were synthesized with relatively high PDIs; the PDI should be improved. Second, all cyclic multimer products were obtained with the PDI values larger than those of their linear PCL precursors. These results are an indication that each cyclic multimer might include impurities (perhaps, coupled linear precursors and cyclic multimers in different sizes) in certain levels. Third, the cyclic multimers inherently include bulky [4+4] cyclized anthracene dimeric units, which can influence their structure and properties. Lastly, this method is still challenged with the task of producing a monomeric cyclization product; any possibility of monomeric cyclization was not shown or discussed.

### 2.3. Intramolecular Cyclizations

#### 2.3.1. Pseudo[2]rotaxane-Initiated Cyclization

**Scheme 3:**
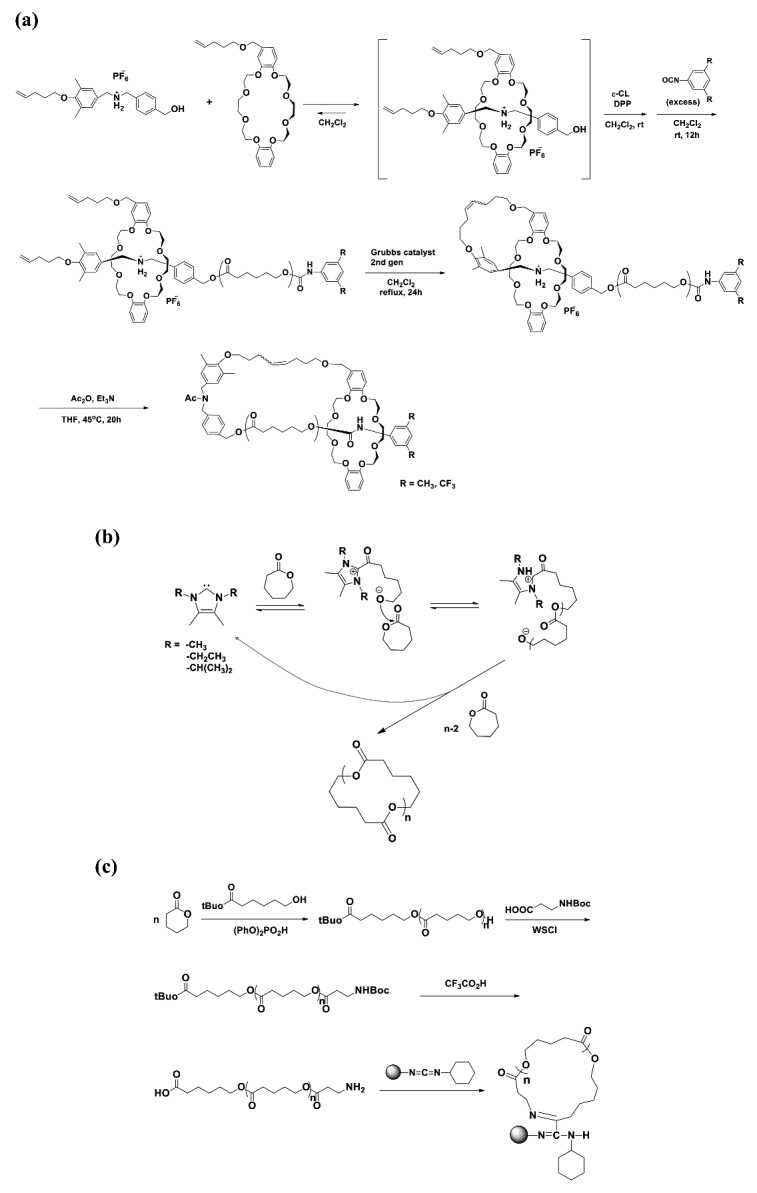
Synthesis of *c*-PCL from CL: (**a**) linear PCL and its cyclization using a rotaxane [10]; (**b**) zwitterionic polymerization of CL [20,21,22]; (**c**) linear PCL and its cyclic amidation [23].

*c*-PCL was synthesized via the ring-opening polymerization of CL initiated by a pseudo[2]rotaxane initiator with the aid of diphenyl phosphate catalyst and subsequent capping of the propagating chain end with a bulky isocyanate (3,5-dimethylphenyl isocyanate or 3,5-bis(trifluoromethyl)phenyl isocyanate) to afford macromolecular [2]rotaxane (Scheme 3a) [10]. Here, the pseudo[2]rotaxane initiator was prepared from *sec*-ammonium salt with both pentenyl and benzyl alcohol termini and dibenzo-24-crown-8-ether possessing pentenyl substituent. The PCL precursor polymers with $\bar{M_{n}}$ = 5500~6700 g/mol were obtained with 75~80% yield and 1.12~1.17 PDI. The successive intramolecular cyclization to macromolecular [1]rotaxane at the precursor polymer terminus was carried out with a yield of 80~82% via the ring-closing metathesis with the aid of Grubbs catalyst (2nd-generation catalyst). Then, the attractive interaction of the terminal ammonium/crown ether moiety was removed via *N*-acetylation; this enabled movement of the crown ether wheel along the axle PCL chain to the urethane region of the other terminus in a solution. The obtained *c*-PCLs showed PDI values very close to those of the linear precursor polymers, indicating that the purification process was done effectively. This approach nicely demonstrated the formation of *c*-PCLs. But, there are several concerns. First, the linear PCL precursors still reveal relatively higher PDI values. Second, the *c*-PCLs possess a very bulky crown ether ring. Such a bulky unit may affect their properties and structure. Lastly, they retain a ring structure based on interactions of the crown ether’s oxygen atoms in the wheel and the amide linker in the axle, which are somewhat weaker than chemical bonds. This non-bond type cyclic coupling may also affect the properties and structure of *c*-PCL.

#### 2.3.2. Zwitterionic Polymerization and Cyclization

*c*-PCL was prepared in THF or toluene at room temperature by the zwitterionic polymerization of CL with an *N*-heterocyclic carbine (NHC) initiator (1,3,4,5-tetramethylimidazol-2-ylidene or 1,3-diethyl-4,5-dimethylimidazol-2-ylidene or 1,3-diisopropyl-4,5-dimethylimidazol-2-ylidene) and subsequent cyclization via intramolecular backbiting of the terminal alkoxides on internal esters of the zwitterions (Scheme 3b) [20,21,22]. The yield of the polymerization with [M]/[I] = 100 was in the range of 30~80% depending on the initiator and time. The *c*-PCL products were determined to reveal $\bar{M_{n}}$ = 41,000~114,000 g/mol and PDI = 1.36~2.16. It is quite novel that this synthetic method can produce *c*-PCLs without any chemical heterogeneities in the ring. However, the polymerization yields are relatively low; and the PDI values are relatively large. There may be three reasons for these results, as follows. The first possibility is that during the polymerization the cyclization reaction, namely intramolecular backbiting of the terminal alkoxides on the internal ester near the cationic terminal, competes with the propagation reactions. The second possible factor is that the intramolecular backbiting of the terminal alkoxide has multiple options on the internal ester units of the growing polymer chain. The third possible factor is that the attack of the terminal alkoxide has more options on the ester linkers of different growing polymer chains.

#### 2.3.3. Cyclic Amidation

*c*-PCL bearing an amide linker was prepared in a four-step reaction (Scheme 3c) [23]. In the first step, linear PCL possessing *α*-*tert*-butoxycarbonyl and *ω*-hydroxyl groups was prepared by the *tert*-butyl-6-hydroxyhexanoate initiated ring-opening polymerization of CL in toluene at room temperature under argon atmosphere with the aid of diphenyl phosphate (DPP) [24] as a metal-free organocatalyst. In the second step, the *ω*-hydroxyl group was reacted with *N*-(*tert*-butoxycarbonyl)-*β*-alanine (*N*-Boc-*β*-alanine), converted to Boc-NH. In the third step, the *α*-*tert*-butoxycarbonyl group was converted to COOH while the *ω*-Boc-NH was converted to NH_2_. In the last step, *α*-COOH-*ω*-NH_2_-PCL was cyclized by a solid-phase amidation method using a silica-supported carbodiimide condensation agent (Silia*Bond*^®^Carbodiimide, Si-DCC) [25,26]. The cyclic amidation was reported to reveal a yield of around 30%. However, no information on the purification and characterization details was given. Thus, serious concerns remain with the *c*-PCL product in terms of purity.

#### 2.3.4. Alkene–Alkene Metathetic Cyclization

**Scheme 4:**
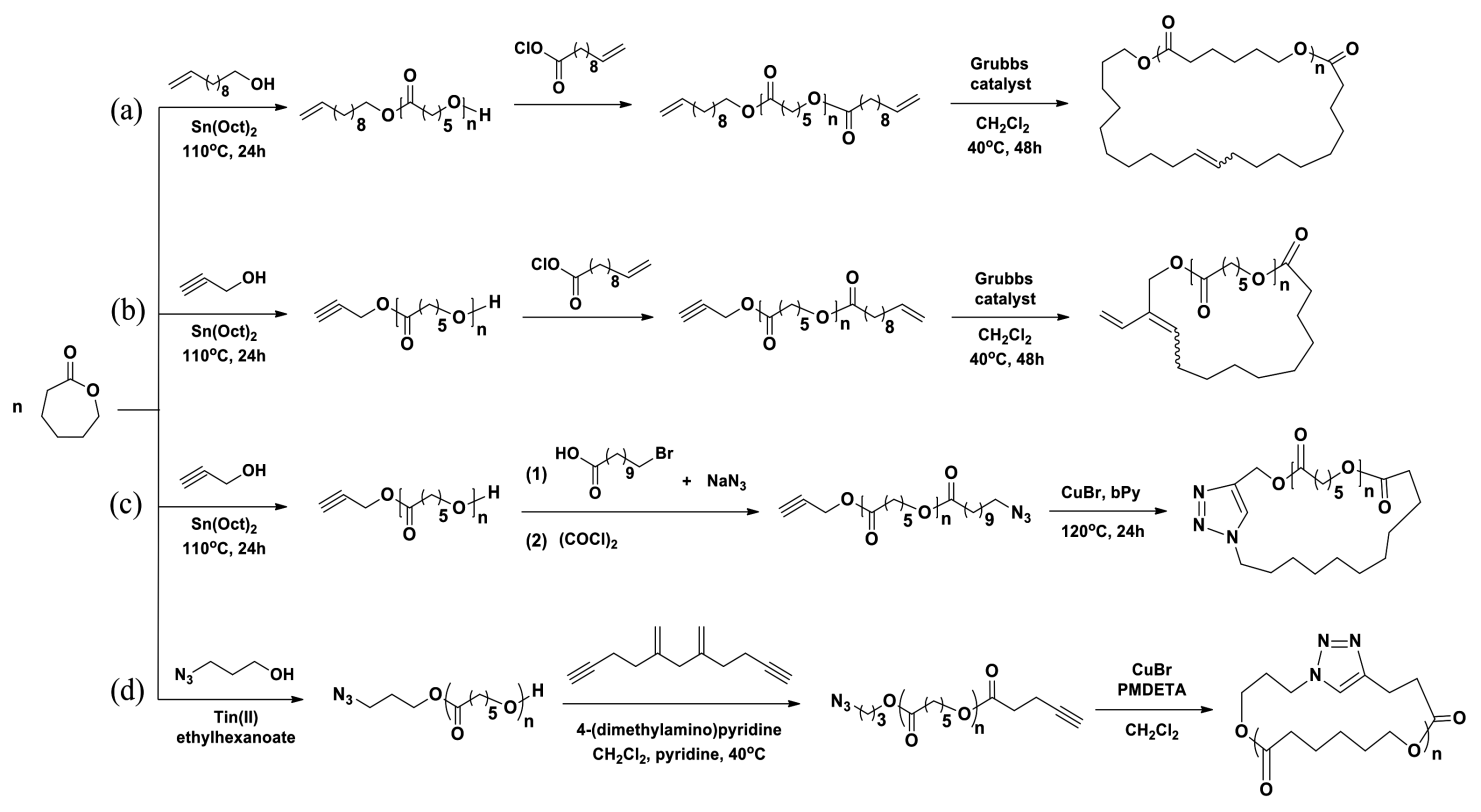
Synthesis of *c*-PCL from CL: (**a**) linear PCL and its cyclization via alkene-alkene metathesis [27]; (**b**) linear PCL and its cyclization via alkyne–alkene metathesis [27]; (**c**) linear PCL and its cyclization via alkyne–azide click chemistry [27]; (**d**) linear PCL and its cyclization via azide–alkyne click chemistry [28].

*c*-PCL was prepared in a three-step reaction (Scheme 4a) [27]. In the first-step, linear *α*-vinyl-*ω*-hydroxy-PCL precursor with $\bar{M_{n}}$ = 3600 g/mol and PDI = 1.08 was prepared in a yield of 75.4% by a polymerization of CL with undecylenyl alcohol initiator and Sn(Oct)_2_ catalyst in toluene at 110 °C for 24 h. The product was purified by precipitation in methanol and subsequent drying. Here it is noted that the *α*-vinyl-*ω*-hydroxy-PCL precursor was obtained with a relatively low PDI value even though Sn(Oct)_2_ was used as catalyst. In the second-step, the hydroxyl end group of the linear precursor was reacted with 10-undecenoyl chloride with the aid of triethylamine in dichloromethane (CH_2_Cl_2_) under a nitrogen atmosphere. The *α*,*ω*-divinyl-PCL product was obtained in a yield of 70%, revealing $\bar{M_{n}}$ = 3900 g/mol and PDI = 1.11. In the third-step, the intramolecular cyclization of *α*,*ω*-divinyl-PCL in a dilute concentration of 0.0005 M was carried out with the aid of Grubbs catalyst (1st-generation catalyst) in CH_2_Cl_2_ at 40 °C for 48 h under a nitrogen atmosphere. The *c*-PCL product with $\bar{M_{n}}$ = 3500 g/mol and PDI = 1.70 was obtained with a yield of 90% after precipitation in methanol, filtering and drying. Overall, oligomeric *c*-PCL product was prepared. For these results, some questions arise as follows. First, the *α*,*ω*-divinyl-PCL was obtained with a relatively low yield. Such low yield suggests that some amount of unreacted *α*-vinyl-*ω*-hydroxy-PCL precursors remained; how could they be eliminated through such simple precipitation process? Second, in the cyclization reaction the *c*-PCL product was obtained with an exceptionally high yield of 90%, but revealing a higher PDI value. Perhaps, the adopted simple precipitation process could not eliminate all possible impurities including unreacted *α*,*ω*-divinyl-PCL precursors. Overall, the results collectively suggest that the *c*-PCL product still includes a certain level of impurities such as unreacted precursors, linear dimers and multimers, and cyclic dimers and multimers. Lastly, it is noted that the *c*-PCL contains an ethenyl linker in the ring which may play as a reactive site for post-chemical modifications. 

#### 2.3.5. Alkyne–Alkene Metathetic Cyclization

In the first-step, linear *α*-ethynyl-*ω*-hydroxy-PCL precursor with $\bar{M_{n}}$ = 4100 g/mol and PDI = 1.09 was prepared in a yield of 77% by a polymerization of CL with propargyl alcohol initiator and Sn(Oct)_2_ catalyst in toluene at 110 °C for 24 h (Scheme 4b) [27]. In the second-step, the *α*-ethynyl-*ω*-hydroxy-PCL precursor was converted to *α*-ethynyl-*ω*-vinyl-PCL through a reaction with 10-undecenoyl chloride under a nitrogen atmosphere; the reaction yield was 72%. The product was measured to have PDI = 1.07. In the final step, the *α*-ethynyl-*ω*-vinyl-PCL precursor in a dilute solution (0.0005 M) underwent cyclization with the aid of Grubbs catalyst (1st-generation catalyst) under a nitrogen atmosphere. The *c*-PCL product was obtained with 89.5% yield after precipitation in methanol, filtering and drying; the product was determined to reveal PDI = 1.6. For these results, some concerns arise as follows. First, the relatively low yield of *α*-ethynyl-*ω*-vinyl-PCL indicates that unreacted *α*-ethynyl-*ω*-hydroxy-PCL precursors could remain in a certain amount. How could such unreacted precursors be removed via the simple precipitation process employed? Second, the yield of *c*-PCL is exceptionally too high in regard to the intramolecular cyclization done in the highly dilute condition. The simple precipitation process was employed to purify the cyclic product; this purification could not completely remove possible unreacted precursors and byproducts from the target product. Therefore, such high product yield might be overestimated, which could be attributed to impurities. Third, the PDI of *c*-PCL was relatively larger than that of the precursor polymer. The larger PDI value again indicates that the *c*-PCL product still included some impurities such as unreacted precursors, linear dimers and multimers, and cyclic dimers and multimers. Lastly, it is noted that the *c*-PCL product possesses two reactive sites: one is the vinyl linker in the ring and another is the vinyl group as a side group. 

#### 2.3.6. Alkyne–Azide Click Cyclization

*c*-PCL was prepared in a three-step reaction (Scheme 4c) [27]. In the first-step, linear *α*-ethynyl-*ω*-hydroxy-PCL precursor with $\bar{M_{n}}$ = 4100 g/mol and PDI = 1.09 was prepared according to the method in Scheme 4c. In the second-step, the *α*-ethynyl-*ω*-hydroxy-PCL precursor was converted to *α*-ethynyl-*ω*-azido-PCL through a reaction with 11-azidoundecanoyl chloride under a nitrogen atmosphere. Yield: 70%. In the final step, the *α*-ethynyl-*ω*-azido-PCL precursor in a dilute solution (0.00067 M precursor concentration) underwent ethyne-azide click cyclization with the aid of copper (I) bromide (CuBr) catalyst and *α*,*α*′-bipyridyl (bPy) in *N*,*N*-dimethylformamide (DMF) at 120 °C for 48 h under a nitrogen atmosphere; the precursor solution was slowly added by using a dropping funnel. The *c*-PCL product was prepared with 91% yield after precipitation in methanol, filtering and drying. The *c*-PCL product was determined to have PDI = 1.1. Overall, the synthesis of *c*-PCL was done in a reasonably good manner. However, some questions still arise as follows. First, the *α*-ethynyl-*ω*-azido-PCL precursor was prepared with a relatively low yield. This result suggests that a certain portion of the *α*-ethynyl-*ω*-hydroxy-PCL precursor remained as an unreacted form; how could a simple precipitation process remove the unreacted precursors from the reaction product? Second, the yield of *c*-PCL product was remarkably high in the consideration of the cyclization reaction in a very dilute solution. And, if the precursors remained in a certain portion as an unreacted form and other polymeric byproducts were generated, they could not easily be removed out through only a simple precipitation process. Thus, the purity of the *c*-PCL product is doubtful. Third, the PDI of *c*-PCL product increased to 1.1 (from 1.03 of the precursor polymer). This increment in the PDI is an indication that the *c*-PCL product still includes polymeric impurities. Lastly, the *c*-PCL product possesses a trizolyl linker in the ring; such planar and bulky triazolyl linker may affect the structure and properties of the cyclic polymer.

#### 2.3.7. Azide–Alkyne Click Cyclization (I)

*α*-Azido-*ω*-hydroxy-PCL precursors were prepared by Sn(Oct)_2_-catalyzed polymerizations of CL with the aid of 3-azidopropanol initiator at 110 °C (Scheme 4d) [28]. The obtained linear precursors were determined to have $\bar{M_{n}}$ = 3830~14,900 g/mol and PDI = 1.07~1.14 depending on the [M]/[I] ratio and reaction time. The *α*-azido-*ω*-hydroxy-PCL precursors were converted to *α*-azido-*ω*-ethynyl-PCLs by the reactions with 4-pentynoic anhydride in CH_2_Cl_2_ at 40 °C with the aids of 4-(dimethylamino)pyridine and pyridine. The *α*-azido-*ω*-ethynyl-PCL products were characterized to reveal PDI = 1.07~1.20. The click cyclizations of the precursors in dilute solutions were conducted in CH_2_Cl_2_ by using CuBr catalyst and *N*,*N*,*N*′,*N*″,*N*″-pentamethyldiethylenetriamine (PMDETA); here each precursor solution (0.0044 M precursor concentration) was added with a rate of 2 mL/h to the CuBr/PMDETA solution by using a syringe pump. After complete addition of the precursor solution, the reaction mixture was stirred for additional 2 h. The reaction mixture was extracted from saturated aqueous NaHSO_4_ into CH_2_Cl_2_. The organic layer was dried over anhydrous MgSO_4_, filtered, and concentrated prior to precipitation from CH_2_Cl_2_ into a 1:1 mixture of chilled hexanes and diethyl ether. The product was isolated via filtration and dried. The yields were around 57%; $\bar{M_{n}}$ = 3780~15,000 g/mol. All *c*-CL products revealed very low PDI values (1.07~1.13), which were comparable to those of the precursor polymers. These results collectively suggest that *c*-PCLs were synthesized with high purity by the azide–alkyne click cyclization. Here, several questions, however, arise about the cyclization reactions and subsequent purification processes as follows. First, the cyclization yields are not high enough (only around 60%), suggesting that each reaction mixture might include a substantial amount of unreacted precursor polymers. Second, how could such unreacted precursors be removed from the *c*-PCL product? Was the employed workup process efficient enough to remove the unreacted precursors out from the *c*-PCL product? Third, could such low PDI values assure purity for the *c*-PCL products? Could each *c*-PCL product have any possibility of including other impurities such as linear dimers and multimers and cyclic dimers and multimers? Lastly, the analytical gel permeation chromatography (GPC) profiles confirmed that the *c*-PCL products include a certain level of impurities even though they are minor components. Thus, the *c*-PCL products still need further purification although they reveal relatively low PDI values.

#### 2.3.8. Azide–Alkyne Click Cyclization (II)

**Scheme 5:**
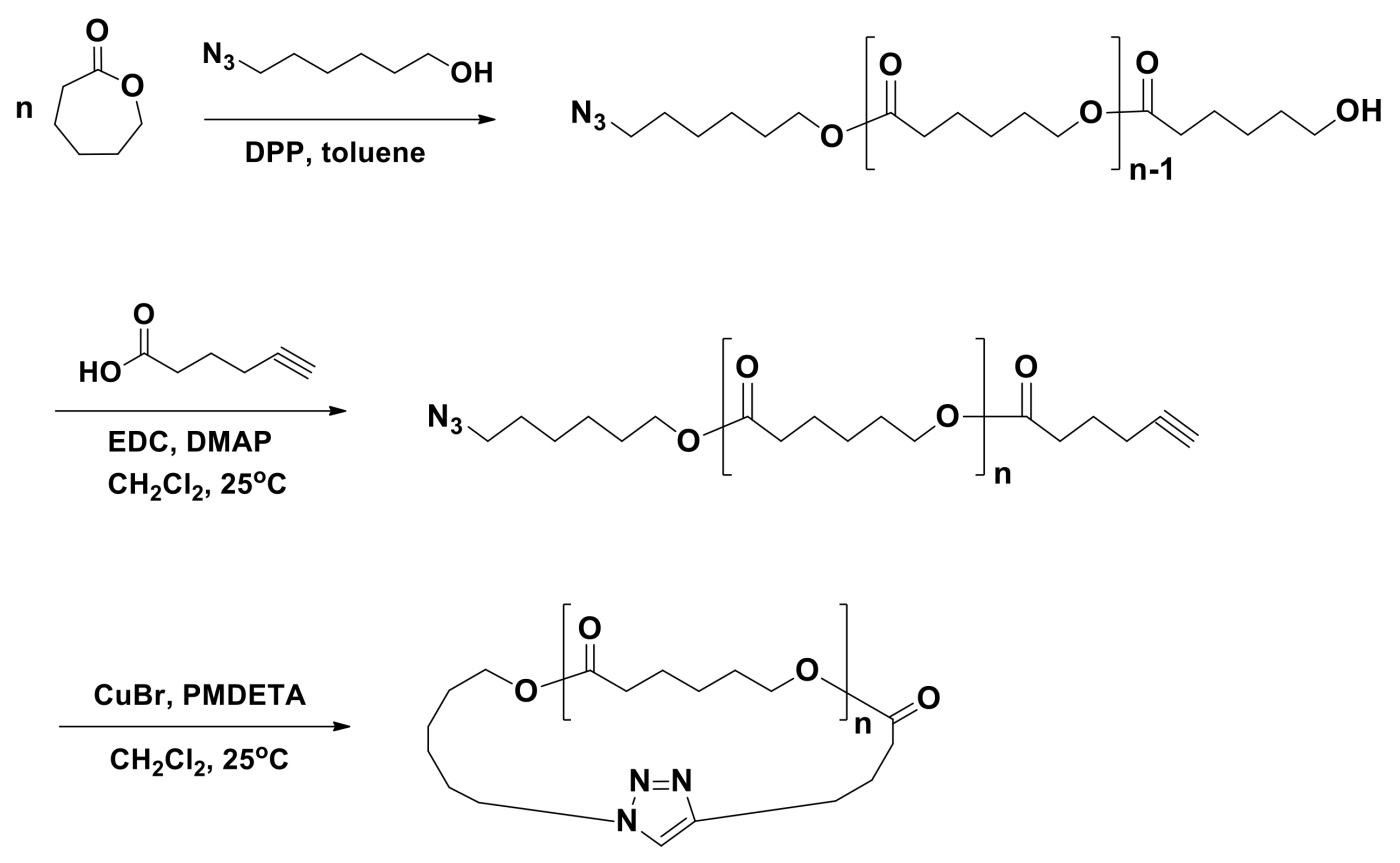
Synthesis of *c*-PCL from CL: linear PCL and its cyclization via azide–alkyne click chemistry.

In this study, we tried to synthesize a series of well-defined, pure *c*-PCLs. Each *c*-PCL was prepared in a three-step manner (Scheme 5). In the first-step, a 6-azido-1-hexanol initiated polymerization of CL was conducted with [M]/[I] = 90 in toluene at 25 °C in a glove box; in this polymerization, diphenyl phosphate was used as an organocatalyst. After 14 h, the polymerization was quenched by adding Amberlyst A21 as a small base bead for termination reaction. After 1 h, the used Amberlyst A21 was removed from the reaction mixture by filtration under a low vacuum filtration and the solvent used was also removed out by using a rotary evaporator with a vacuum system. The crude product was dissolved in CH_2_Cl_2_ and then precipitated into cold methanol, followed by filtration. The product obtained was washed with cold methanol several times and then dried at room temperature in vacuum. The *α*-azido-*ω*-hydroxy-PCL (N_3_-PCL_90_-OH) precursor product was obtained with 96.8% yield. The N_3_-PCL_90_-OH product was characterized by proton (^1^H) and carbon (^13^C) nuclear magnetic resonance (NMR) spectroscopies; a Bruker spectrometer (model AV300 FT-NMR, Rheinstetten, BW, Germany) was employed. ^1^H NMR (300 MHz, CDCl_3_, δ (ppm)): 1.38 (m, 2H × *n*, (–CH_2_CH_2_CH_2_CH_2_CH_2_–)*_n_*), 1.57 (m, 2H × *n*, (−CH_2_CH_2_CH_2_O−)*_n_*), 1.65 (m, 2H × *n*, (−COCH_2_CH_2_CH_2_−)*_n_*), 2.31 (t, 2H × *n*, (−OCOCH_2_CH_2_−)*_n_*), 3.28 (t, 2H, N_3_CH_2_−), 3.65 (t, 2H, −CH_2_OH), 4.06 (t, 2H × *n*, (−CH_2_CH_2_O−)*_n_*). ^13^C NMR (150 MHz, CDCl_3_, δ (ppm)): 24.5, 24.7, 25.0 ((−CH_2_CH_2_CH_2_−)*_n_*), 26.4, 28.9, 30.1 (−CH_2_CH_2_CH_2_−), 31.9 (−CH_2_CH_2_OH), 33.9 (−OCOCH_2_−), 51.3 (N_3_CH_2_−), 62.5 (−CH_2_CH_2_OH), 64.1 (−CH_2_CH_2_OCO−), 173.5 (−OCOCH_2_−). The number-average molecular weight of the product was determined as calculated by proton ^1^H NMR spectroscopy analysis: ${\bar{M_{n,}}}_{NMR}$ = 10,100 g/mol. The product was further characterized by using a GPC system (Waters Alliance e2695 GPC system, Milford, MA, USA) equipped with Styragel^®^ columns (HR1, HR2 and HR4), Waters 1515 isocratic pump and 2414 refractometer): PDI = 1.09. In the same manner, the other *α*-azido-*ω*-hydroxy-PCL precursors were prepared by the polymerizations with changing [M]/[I] ratio and time, followed by purifications and characterizations. 

In the second-step, N_3_-PCL_90_-OH was further reacted with 5-hexynoic acid in CH_2_Cl_2_ at 25 °C under a nitrogen atmosphere by the aids of 1-ethyl-3-(3-dimethylaminopropyl)carbodiimide (EDC) hydrochloride and 4-(dimethylamino)pyridine (DMAP). After 48 h, the reaction mixture was concentrated by using a rotary evaporator with a low vacuum system. The crude product was dissolved in CH_2_Cl_2_ and then precipitated into cold methanol several times to remove residual 5-hexynoic acid, EDC and DMAP, followed by filtration. The obtained product (N_3_-PCL_90_-C≡CH) was washed with cold methanol several times and dried at room temperature in vacuum. Yield: 98.1%. ${\bar{M_{n,}}}_{NMR}$ = 10,200 g/mol; PDI = 1.10. ^1^H NMR (300 MHz, CDCl_3_, δ (ppm)): 1.38 (m, 2H × *n*, (−CH_2_CH_2_CH_2_CH_2_CH_2_−)*_n_*), 1.57 (m, 2H × *n*, (−CH_2_CH_2_CH_2_O−)*_n_*), 1.85 (quin, 2H, −CH_2_CH_2_C≡C), 1.98 (t, H, −C≡CH), 2.27 (m, 2H, −CH_2_C≡C), 2.31 (t, 2H × *n*, (−OCOCH_2_CH_2_−)*_n_*), 2.46 (t, 2H, −CH_2_CH_2_CH_2_C≡C), 3.28 (t, 2H, N_3_CH_2_−), 4.06 (t, 2H × *n*, (−CH_2_CH_2_O−)*_n_*). ^13^C NMR (150 MHz, CDCl_3_, δ (ppm)): 17.3 (CH_2_C≡CH), 23.9, 24.5, 24.7, 25.0 ((−CH_2_CH_2_CH_2_−)*_n_*), 26.4, 28.9, 30.1 (−CH_2_CH_2_CH_2_−), 31.9 (−CH_2_CH_2_OH), 32.7, 33.9 (−OCOCH_2_−), 51.3 (N_3_CH_2_−), 64.2 (−CH_2_CH_2_OCO−), 69.0 (−C≡CH), 83.5 (−CH_2_CC≡H), 173.4 (−OCOCH_2_−). In the same manner, the other *α*-azido-*ω*-ethynyl-PCLs were prepared, purified, and characterized.

**Figure 1:**
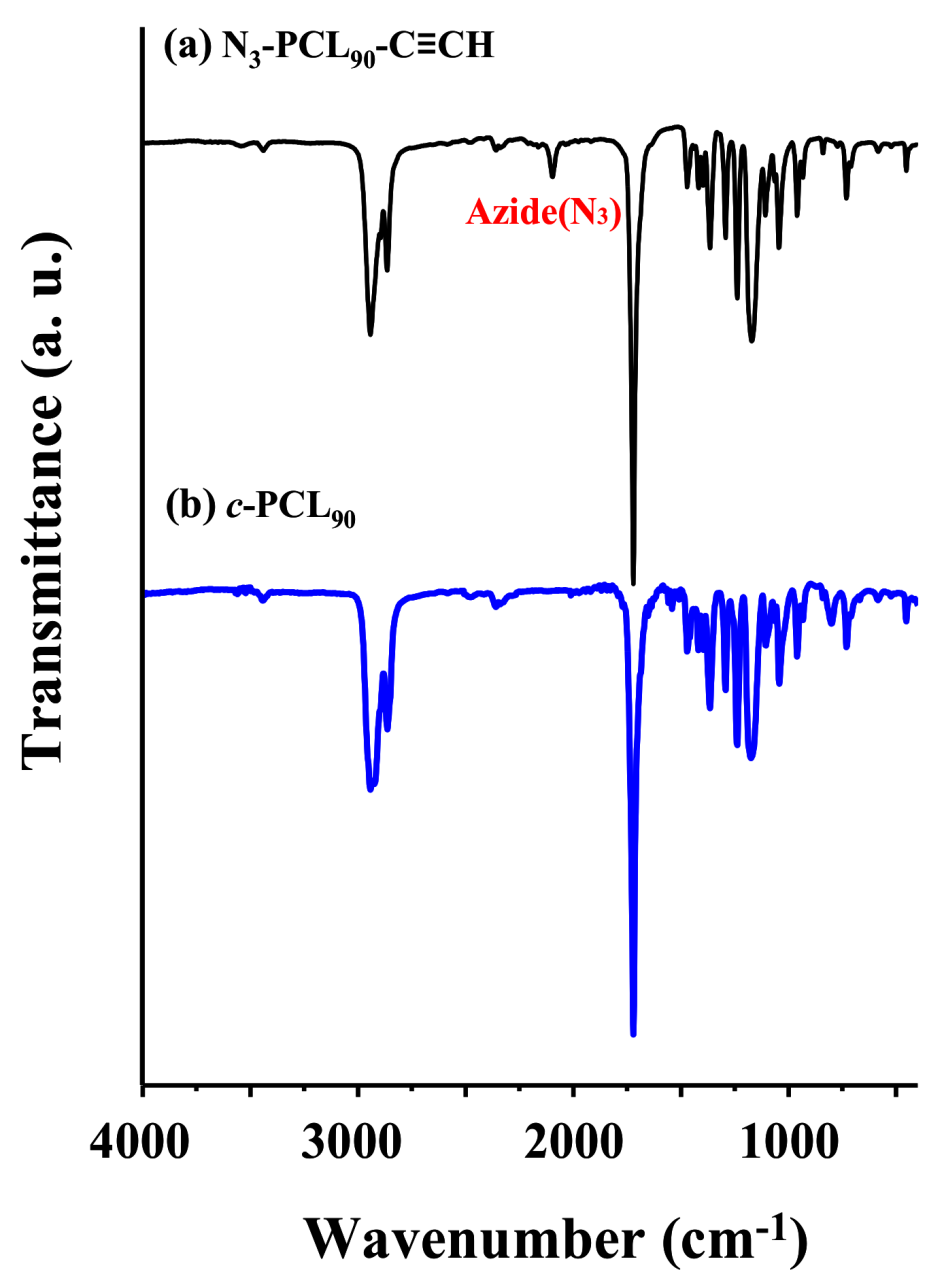
Fourier transform–infrared (FT–IR) spectra of a linear PCL precursor and its cyclic product.

In the third-step, N_3_-PCL_90_-C≡CH was cyclized as follows. The N_3_-PCL_90_-C≡CH (510 mg, 15.5 μmol) in degassed CH_2_Cl_2_ (36 mL) (0.00043 M precursor concentration) was added with a rate of 0.5 mL/h to a mixture of CuBr (386 mg, 0.837 mmol) and PMDETA (0.68 mL, 1.01 mmol) in degassed CH_2_Cl_2_ (350 mL) at 25 °C under flowing argon using a syringe pump (model Legato 100, KD Scientific, Holliston, MA, USA) equipped with a fine hypodermic needle (21 g × 30 cm). After the addition was completed, the reaction mixture was stirred for additional 3 h. Then, propargyl-functionalized polystyrene (PS-C≡CH) resin (2.5 g, 7.35 mmol) and a solution of CuBr (772 mg, 0.837 mmol) and PMDETA (1.36 mL, 1.01 mmol) were added in order to further reactions with unreacted precursor polymers as well as possibly formed linear dimers and multimers; here, PS-C≡CH resin was prepared by the treatment of 4-(hydroxymethyl)phenoxymethylpolystyrene resin (polystyrene resin cross-linked with 1% divinylbenzene (200–400 mesh)) with propargyl bromide [29,30]. After being stirred for 24 h, the reaction mixture was filtered, eliminating the unreacted precursors and possible linear dimers and mutlimers together with the used PS resin. The filtrate was concentrated by using a rotary evaporator. The crude product was purified by using aluminum oxide columns (eluent, THF). The obtained product was further purified using a preparative GPC system, followed by drying at room temperature under vacuum. The recycling preparative GPC runs were carried out with THF (7.5mL/min) at 25 °C using a JAI GPC system (model LC-9260 II Next, Japan Analytical Industry, Tokyo, Japan) equipped with a JAI JAIGEL-2.5HH column (600 × 20.0 mm), a JAI JAIGEL-2HH column (600 × 20.0 mm) and a JAI RI-700 II NEXT refractive index detector. The target cyclic product (*c*-PCL_90_) was obtained. Yield: 38.9%. The *c*-PCL_90_ product was characterized by NMR spectroscopy and GPC. ${\bar{M_{n,}}}_{NMR}$ = 10,200 g/mol; PDI = 1.08. ^1^H NMR (300 MHz, CDCl_3_, δ (ppm)): 1.38(m, 2H × *n*, (−CH_2_CH_2_CH_2_CH_2_CH_2_−)*_n_*), 1.57(m, 2H × *n*, (−CH_2_CH_2_CH_2_O−)*_n_*), 2.31 (t, 2H × *n*, (−OCOCH_2_CH_2_−)*_n_*), 2.76 (t, 2H, −CH_2_C=CH−), 4.06(t, 2H × *n*, (−CH_2_CH_2_O−)*_n_*), 4.43 (t, 2H, NCH_2_−), 7.31 (s, 1H, triazole ring). ^13^C NMR (150 MHz, CDCl_3_, δ (ppm)): 24.4, 24.7, 25.1 ((−CH_2_CH_2_CH_2_−)*_n_*), 26.0, 26.4, 29.9, 30.1, (−CH_2_CH_2_CH_2_−), 33.6 (−OCOCH_2_−), 57.0 (−CH_2_CH_2_N−), 66.7 (−CH_2_CH_2_OCO−), 120.2 (triazole ring), 146.8 (triazole ring), 173.5(−OCOCH_2_−). The *c*-PCL_90_ product was further confirmed by using an infrared (IR) spectrometer (model Research Series 2, ATI Mattson, Madison, WI, USA) equipped with a liquid nitrogen-cooled mercury cadmium telluride (MCT) detector (Figure 1). In the same manner, cyclization reactions were conducted for the other *α*-azido-*ω*-ethynyl-PCLs, then followed by purifications and characterizations.

As described above, we adopted diphenyl phosphate as a metal-free, nontoxic organocatalyst and 6-azido-1-hexanol as a initiator for the ring opening polymerization of CL. Linear PCL products were obtained with very high yields (96.5~97.1%) over the range [M]/[I] = 20~200 and determined to reveal very low PDI values (1.06~1.10), as shown in Table 1. The high yields and low PDI values are much better than those of the PCL products obtained by the Sn(Oct)_2_-catalyzed polymerizations [27,28]. The yield and PDI results are further improved, compared to those of the pseudo[2]rotaxane-initiated polymerizations with the aid of diphenyl phosphate catalyst [10]. These collectively inform the following key features. First, for the polymerization of CL, diphenyl phosphate is a better catalyst in the aspect of catalytic performance, compared to Sn(Oct)_2_. Moreover, diphenyl phosphate is a non-metallic catalyst and, therefore, suitable to produce linear PCLs free of metallic residues. Second, chemical characteristics and bulkiness of a chosen initiator can have a significant influence on the yield, molecular weight and molecular weight distribution of PCL product.

The obtained *α*-azido-*ω*-hydroxyl-PCLs were fully converted to the *α*-azido-*ω*-ethynyl-PCLs via the reactions with 5-hexynoic acid in excess. The product yields ranged from 96.8 to 98.1% (Table 2). In fact, the reaction conversion was 100% for each reaction; some losses of the individual products took place through the purification processes.

**Figure 2:**
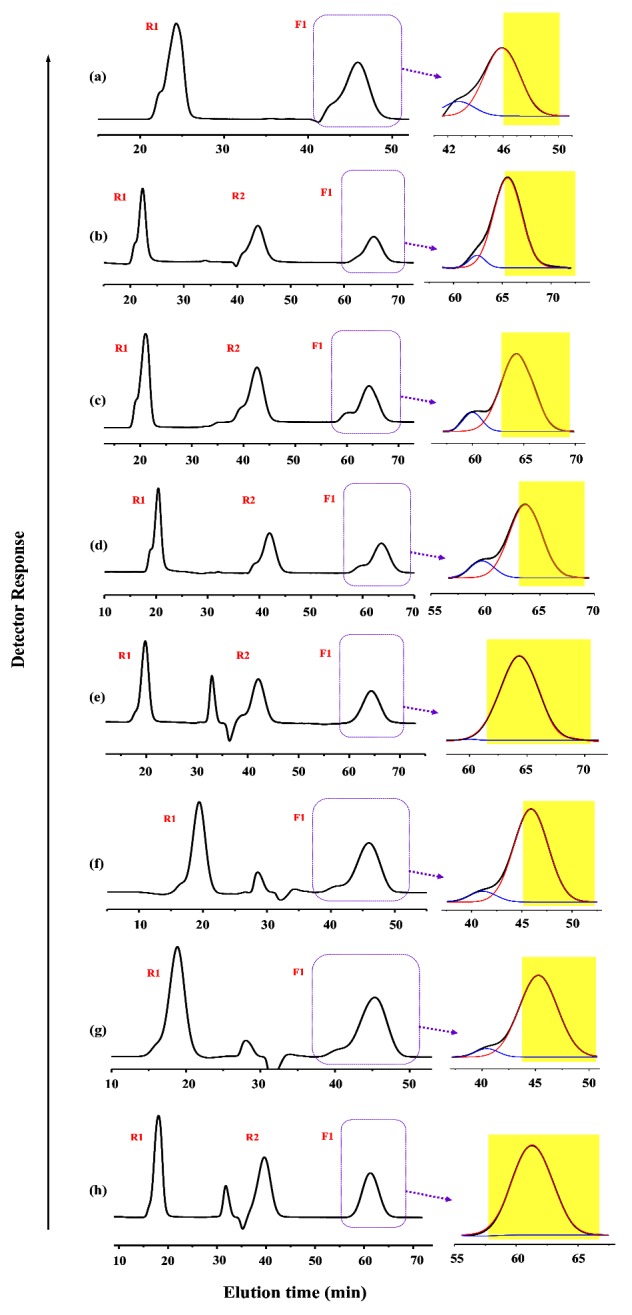
Separation and purification of *c*-PCL using a recycling preparative GPC system: (**a**) *c*-PCL_20_; (**b**) *c*-PCL_30_; (**c**) *c*-PCL_50_; (**d**) *c*-PCL_70_; (**e**) *c*-PCL_90_; (**f**) *c*-PCL_100_; (**g**) *c*-PCL_150_; (**h**) *c*-PCL_200_. R1 and R2 indicate the first and second cycling runs respectively in the GPC operation. F1 means the fractionation of *c*-PCL product; the *c*-PCL product marked with yellow color was fractionated.

**Figure 3:**
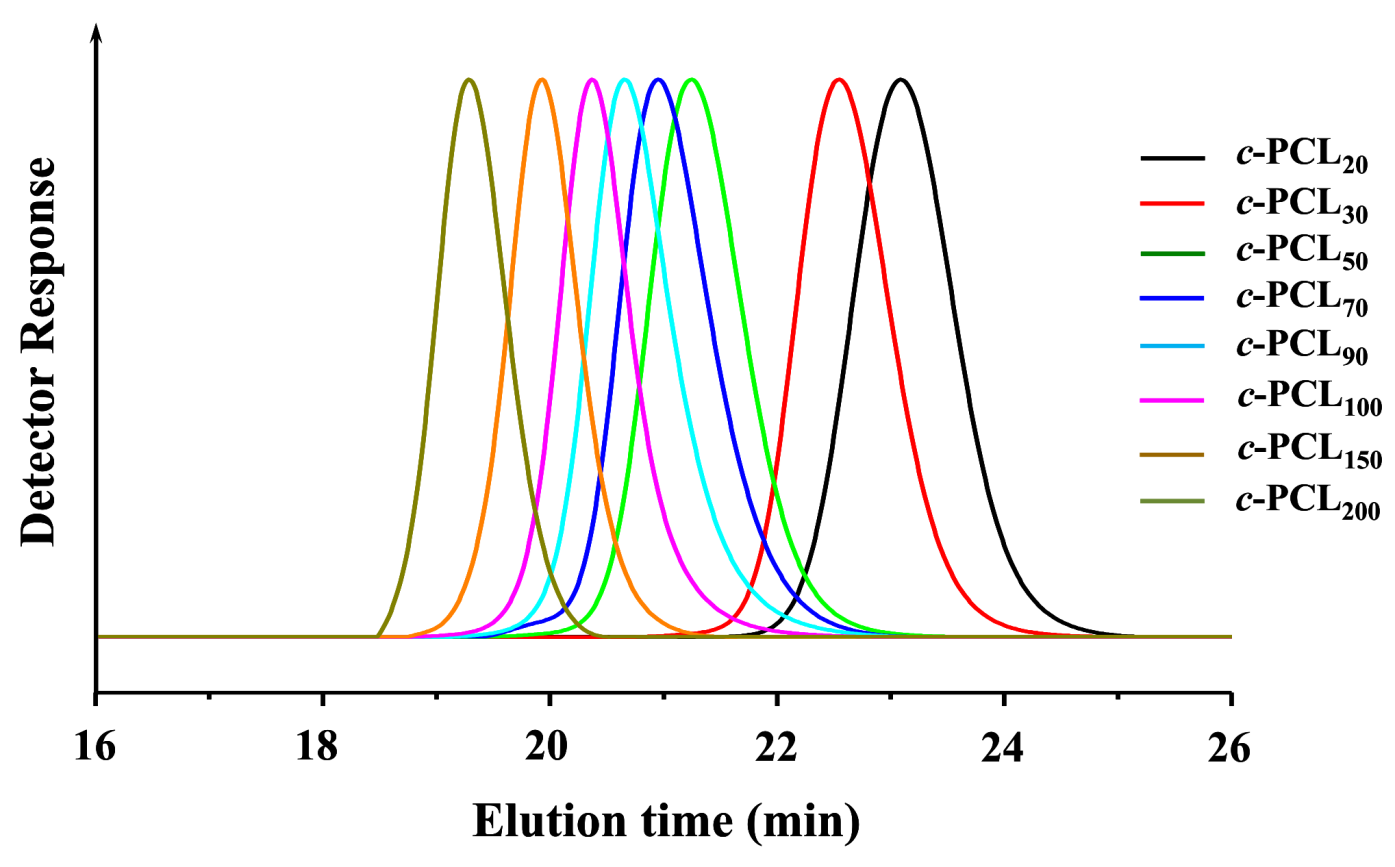
Analytical GPC chromatograms of the *c*-PCLs separated and fractionated (i.e., purified) by using preparative GPC (Figure 2).

For the *α*-azido-*ω*-ethynyl-PCLs, intramolecular cyclizations were attempted via azide–alkyne click chemistry with the aid of CuBr and PMDETA. Similar cyclizations were previously done with a precursor concentration of 0.00067~0.00440 M; a dropping funnel and a syringe pump system were employed to add the precursor solutions in a precisely-controlled dropping manner [27,28]. In the case of the syringe pump system, an injection rate of 2 mL/h was used [28]. In these studies simple precipitation methods were employed to purify cyclic products. In view of the high precision and control in the addition of precursor solutions, a digital-based syringe pump system would be much better than a dropping funnel. To get *c*-PCL of high or highest purity, a high-performance purification process, rather than simple precipitation methods, is absolutely necessary. Therefore, in our study, we decided to adopt the use of highly dilute precursor solution (0.00043 M), a high-precision syringe pump system with a very fine needle for the precisely-controlled addition of precursor solution (0.5 mL/h injection rate), a PS-C≡CH resin treatment to remove unreacted precursors and linear byproducts and subsequent filtration, a chromatographic treatment to remove the used catalyst, and a recycling preparative GPC system to purify the target cyclic product, as described above (Figure 2). The purified *c*-PCL products were further characterized by using an analytical GPC system calibrated with polystyrene standards (Figure 3).

The *c*-PCL products were obtained with yields ranging from 37.7 to 41.1%; the cyclic byproducts were additionally formed with yields ranging from 0.2 to 8.7% (Figure 2; Table 2 and Table 3). The yield data for the *c*-PCL products are somewhat scattered. The cyclic byproducts’ yields are also scattered. However, the *c*-PCL products show a trend that the product yield increased slightly by increasing the molecular weight of precursor polymer. In contrast, the cyclic byproducts exhibit a trend that their yield decreased with increasing the molecular weight of precursor polymer. On the other hand, the total amount of unreacted precursors and their linear dimmer and multimers ranges from 52.2 to 60.9%. These results collectively provide important information on the intramolecular cyclization reaction via azide–alkyne click reaction as follows.

First, the generation of byproducts could not be avoided even under cyclization reactions in highly dilute concentration (0.00043 M, which is the lowest concentration in comparison to those reported in the literature) and very slow addition (0.5 mL/h) with very small drop size (which are the smallest drop size and slowest addition, compared to those reported in the literature).

Second, a target cyclic product could be obtained with relatively low yield, which is attributed to an unavoidable amount of unreacted precursors remaining due to the very dilute precursor concentration in the reaction as well as byproduct formations.

Third, cyclic byproducts could be formed in much lower fractions, compared to the sum of unreacted precursors and linear byproducts (dimers and multimers).

Fourth, the unreacted precursor polymers and linear byproducts could be eliminated effectively by their reactions with alkyne- or azide-functionalized polymer resins and subsequent filtration. Their removals could not be easy or possible only through a conventional, simple precipitation process.

Fifth, the removal of cyclic byproducts could not be easy. They could be removed via tedious, labor-intensive separation and fractionation by using a preparative or analytical chromatography system. Their removals are also impossible only through a conventional, simple precipitation process.

Lastly, the intramolecular azide–alkyne click cyclization method still finds it challenging to increase target cyclic product yield and reduce byproducts even though the generally-known high efficiency of azide–alkyne click chemistry. Moreover, effective elimination of unreacted precursors and byproducts is essential to get the desired cyclic polymer product in high purity. To obtain a higher quality cyclic polymer (namely, cyclic product with a lowest PDI), the synthesis of higher quality linear precursor polymer is absolutely necessary.

## 3. Chain Structure Characteristics

Information on chain structure characteristics is necessary to understand *c*-PCL itself and its structure and properties at a molecular level and to develop its applications. However, the chain characteristics of *c*-PCL have been investigated on a very limited basis. The chain characteristics of *c*-PCL evident in hydrodynamic volume, solution viscosity, melt viscosity and chain mobility in the melt state are reviewed below.

### 3.1. Hydrodynamic Volume

**Figure 4:**
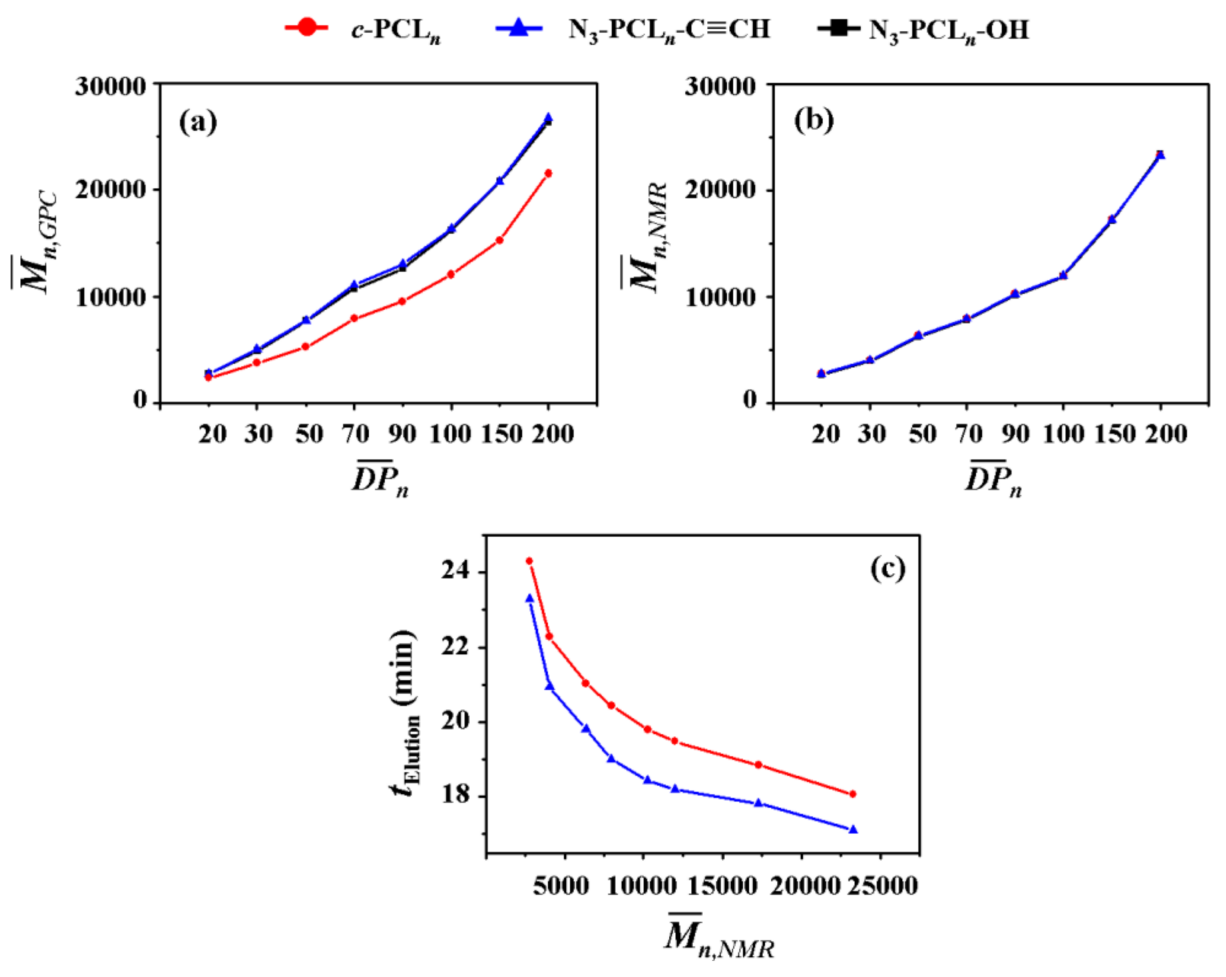
GPC and ^1^H NMR spectroscopy analysis results of *c*-PCLs and their linear analogues: (**a**) number-average degree of polymerization (${\bar{DP}}_{n}$) versus ${\bar{M_{n,}}}_{GPC}$; (**b**) ${\bar{DP}}_{n}$ versus ${\bar{M_{n,}}}_{NMR}$; (**c**) elution time (GPC) versus ${\bar{M_{n,}}}_{NMR}$. ${\bar{M_{n,}}}_{GPC}$: number-average molecular weight determined by a GPC system calibrated with polystyrene (PS) standards. ${\bar{M_{n,}}}_{NMR}$: number-average molecular weight determined by ^1^H NMR spectroscopy analysis.

GPC is widely used to determine the ${\bar{M_{n,}}}_{GPC}$ and PDI (=${\bar{M_{w,}}}_{GPC}$/${\bar{M_{n,}}}_{GPC}$; ${\bar{M_{w,}}}_{GPC}$, weight-average molecular weight determined by GPC analysis) of polymer with respect to those of standard polymers (linear polystyrene (PS) or polymethylmethacrylate (PMMA) standards employed widely). GPC is also a good analytical tool to provide information on the geometrical dimensions of *c*-PCLs in addition to the determination of molecular weights and PDI because of its size-exclusion ability. In this study, we have conducted GPC analysis on the series of *c*-PCLs and their linear precursors synthesized with high purity in the section above; here the GPC system has been calibrated with linear PS standards rather than cyclic PS standards because cyclic PS standards are not available, and THF was used as an eluent. The individual N_3_-PCL*_n_*-C≡CH polymers reveal ${\bar{M_{n,}}}_{GPC}$ values almost same with those of their N_3_-PCL*_n_*-OH precursors, as shown in Figure 4a (also data available in Table 1 and Table 2). These results support the fact that the individual N_3_-PCL*_n_*-C≡CH polymers apparently have same hydrodynamic volumes in THF solutions as their N_3_-PCL*_n_*-OH precursors exhibit even though there is a difference in the *ω*-functional groups.

In contrast, the individual *c*-PCL products exhibit relatively smaller ${\bar{M_{n,}}}_{GPC}$ values than those of their N_3_-PCL*_n_*-C≡CH precursors (Figure 4a and Table 2) even though they have molecular weights (${\bar{M_{n,}}}_{NMR}$ values) the same as those of their precursors (Figure 4b and Table 2). The *c*-PCL products reveal longer elution times (*t*_elution_), compared to those of the linear counterparts (Table 4c). Similar GPC analysis results were reported for the *c*-PCLs and their linear analogues in the literature [10,16,17,18,27,28,31,32,33,34,35]; however, it is noted that the *t*_elution_ differences were influenced by the impurity levels in the *c*-PCLs. Overall, the GPC analysis results confirm that *c*-PCLs have relatively smaller hydrodynamic volumes in comparison to their linear analogues. Such dynamic volume difference due to the cyclic topology tends to be increased as the molecular weight increases.

### 3.2. Solution Viscosity

A series of *c*-PCLs and their linear analogues have been characterized as dilute solutions in THF by viscometry combined with a GPC system equipped with light scattering [18]; here all *c*-PCLs were synthesized by using a ring-expansion method with the aids of alkyl-organoaluminum initiators. The intrinsic viscosity [*η*] was increased with molecular weight for the *c*-PCLs as well as the linear PCL analogues. In comparison, the invidual *c*-PCLs always showed lower viscosities than those of their linear counterparts. These results again prove that *c*-PCLs have smaller hydrodynamic volumes relative to those of their linear counterparts. These are further correlated to the GPC analysis results discussed above.

### 3.3. Melt Viscosity and Chain Mobility

Melt rheology analysis was carried out on *c*-PCLs and their linear counterparts with ${\bar{M_{n,}}}_{GPC}$ = 63,400 g/mol (1.6 PDI) and 69,200 g/mol (1.8 PDI) at 60 and 80 °C; here the *c*-PCLs were prepared by ring-expansion polymerizations with the aid of a stannane initiator [31]. This analysis found that the melt viscosities *η* of the *c*-PCLs are lower than those of the linear analogues by nearly a factor of 2; even considering a low level of impurities, such lower *η* values of the *c*-PCLs might result from their shear motions (i.e., chain segmental motions; molecular diffusions) being much easier than those of the linear analogues. Such higher segmental mobilities of *c*-PCLs in the melt state were confirmed by NMR Hahn echo and more advanced multiquantum analysis [31].

In fact, linear PCLs were intensively investigated in the view of melt rheology [35,36,37,38,39,40]. Two research groups found that the critical molecular weight *M_e_* for the development of molecular entanglements in linear PCL is close to $\bar{M_{w}}$ = 2000~6000 g/mol [35,36,37,38,39]. However, another research group identified that the linear PCLs of $\bar{M_{w}}$ = 2000~7000 g/mol still are not in completely free of entanglements [35,40]. Linear PCL is further known to have a Kuhn segment length of 0.7 nm [41]. A similar Kuhn length is expected for *c*-PCL. In fact, the cyclic topology may however cause a certain degree of geometrical confinement on the chain conformation even its impact is low and, therefore, a little bit larger Kuhn length may be expected for *c*-PCL. Taking into consideration these, the relatively lower melt viscosities of *c*-PCLs suggest that *c*-PCL reveals relatively larger *M_e_* than that of its linear counterpart. Furthermore, *c*-PCLs of >2000 g/mol $\bar{M_{w}}$ may behave with a relatively lower degree of entanglements, compared to their linear analogues. These interesting features would obviously originate from the cyclic topology of *c*-PCLs in free of end-groups. However, more research is still necessary to understand more about chain characteristics of *c*-PCLs in the melt.

## 4. Properties

A research effort has been made to understand the properties of *c*-PCL. However, such research efforts have been mainly focused on hydrolysis, thermal degradation, and physical phase transitions. These properties are reviewed in this section. In addition, we discuss new results on the thermal degradation and phase transitions which we have measured for the series of *c*-PCL products and their linear analogues synthesized in high purity as discussed above.

### 4.1. Acid–Catalytic Degradation

The synthesis of *c*-PCL was studied in relatively intensive way as discussed above. On the contrary, its catalytic degradation behavior was vey rarely investigated. For example, the degradation characteristics of a *c*-PCL with ${\bar{M_{n,}}}_{MALDI-TOF}$ = 6180 g/mol and its linear azide-ethynyl precursor were examined in a methanol/dichloromethane mixture at room temperature by the aid of toluenesulfonic acid as a catalyst [28]. Samplings were conducted with degradation time intervals and characterized by GPC and matrix-assisted laser desorption time-of-flight mass spectroscopy (MALDI-TOF MS). Overall, *c*-PCL reveals relatively slower degradation behavior than the linear precursor polymer. In particular, *c*-PCL showed significant retardation in the early stages of degradation (up to ca. 3 h). Such retarded degradation could be attributed to the opening of the ring structure, evidencing the effect of cyclic topology in the free chain ends.

### 4.2. Thermal Degradation

Only one report on the thermal degradation of *c*-PCL is available in the literature; two *c*-PCLs with different molecular weights (${\bar{M_{n,}}}_{MALDI-TOF}$ = 4940 and 15,000 g/mol) were investigated [28]. The study concluded that the cyclic topology does not have a significant effect on thermal degradation. Overall, the thermal degradation behavior of *c*-PCL has been very rarely studied, compared to its synthesis as well as its linear forms; a greater understanding of the cyclic topology effect on the thermal degradation of PCL is required.

**Figure 5:**
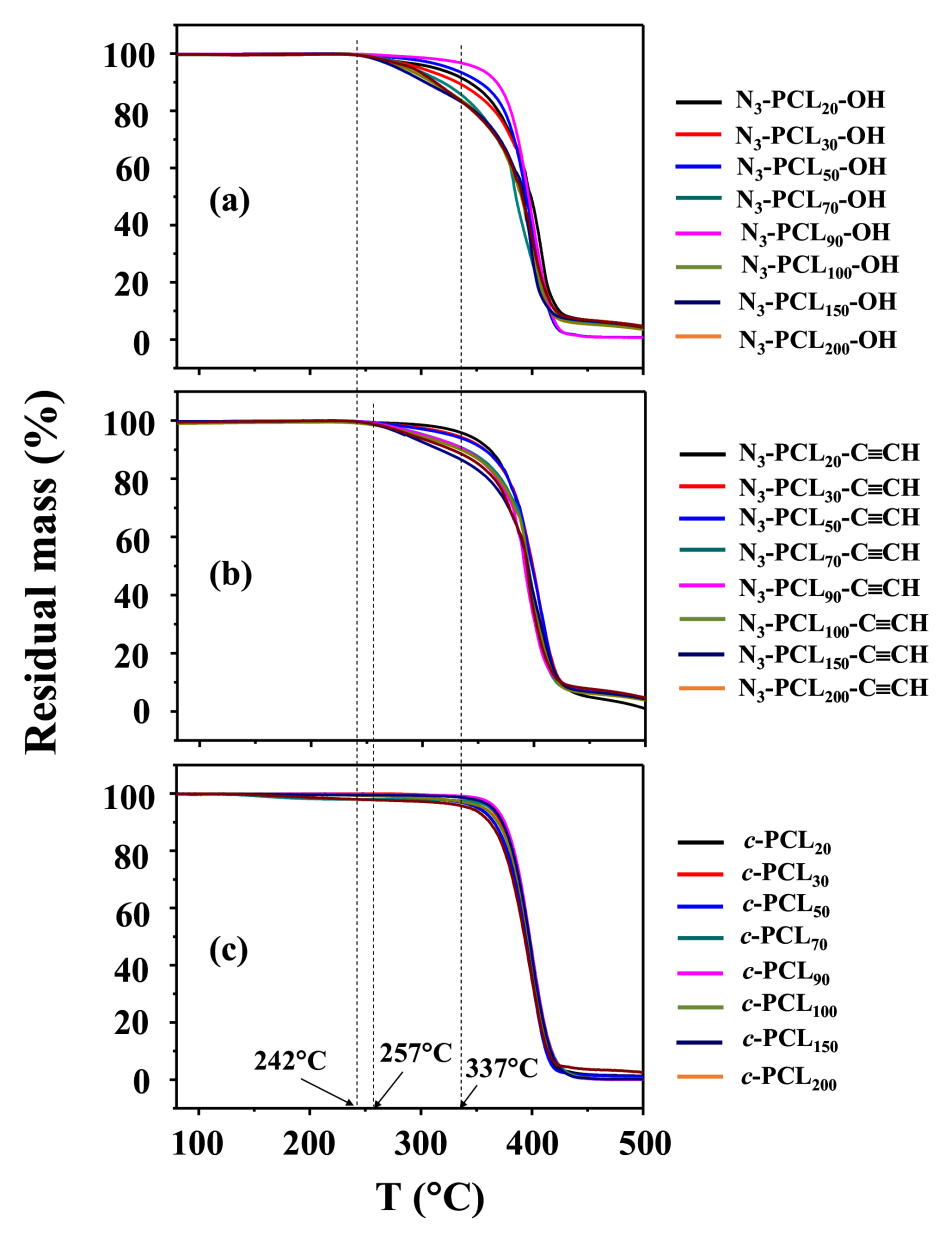
Thermogravimetric analysis (TGA) of *c*-PCLs and their linear analogues. The measurements were conducted with a rate of 10.0 °C/min under nitrogen atmosphere.

Therefore, we conducted TGA analysis on the series of *c*-PCLs with different molecular weights and their linear precursors synthesized above (Table 1 and Table 2). The N_3_-PCL*_n_*-OH precursors (${\bar{M_{n,}}}_{NMR}$ = 2680~24,300 g/mol) started weight loss around 242 °C and continued weight losses in a minor manner, then followed by major weight losses above 337 °C (Figure 5a). Namely, they revealed a two-step manner of thermal degradation under a nitrogen atmosphere: *T*_d1,onset_ = 242 °C (the onset temperature of the first-step degradation) and *T*_d2,onset_ = 337 °C (the onset temperature of the second-step degradation). Here, it is noted that the first-step degradation took place weakly and thus was discernible for some precursors, but not clearly discernible for the other precursors because of continuous weight loss behaviors. In comparison, a major degradation occurred in the second-step. Similar degradation characteristics were previously observed for linear *α*-azido-*ω*-hydroxyl-PCLs (${\bar{M_{n,}}}_{MALDI-TOF}$ = 4940 and 15,000 g/mol) [28] and also for linear *α*-isopropyl-*ω*-hydroxyl-PCLs (${\bar{M_{n,}}}_{osmometry}$ = 1800~42,450 g/mol) [42]. The TGA analysis coupled with mass and IR spectroscopies confirmed the first-step degradation results from a statistical rupture of the ester backbone chains via ester pyrolysis reaction, while the second-step degradation is led by the formation of CL monomers as a result of unzipping depolymerization process [42]. Considering these facts, our N_3_-PCL*_n_*-OH precursors may follow a similar mechanism during thermal degradation, regardless of molecular weight. Taking these facts into account further, weight-loss signals in the first-step degradation could be dependent upon how many volatile fragments are generated by such pyrolysis-induced chain rupturing. Indeed, our results show that relatively small amounts of volatile fragments were generated in the first-step event and their amounts varied with the precursor’s molecular weights in an irregular manner.

Similar two-step degradation behaviors were observed for the N_3_-PCL*_n_*-C≡CH precursors even though the *ω*-hydroxyl end was capped by 5-hexynoic acid in addition to the azido-capped chain end (Figure 5b). These results suggest that the N_3_-PCL*_n_*-C≡CH precursors followed a thermal degradation mechanism similar to that of N_3_-PCL*_n_*-OH precursors. Interestingly, the precursors showed higher *T*_d1,onset_ (257 °C) but similar *T*_d2,onset_, compared to those of their starting polymers (N_3_-PCL*_n_*-OH). These results confirm that the thermal stability of linear PCL precursor polymer was improved by ethynyl-capping of one chain end in addition to azido-capping of the other chain end. Overall, these results are different from those claimed previously in the literature [28].

The *c*-PCL polymers exhibited a thermal degradation in a single-step manner rather than a two-step manner, regardless of molecular weights (Figure 5c). The onset degradation temperature was 337 °C, which was same with the *T*_d2,onset_ values of their N_3_-PCL*_n_*-C≡CH and N_3_-PCL*_n_*-OH precursors. These TGA results indicate that the *c*-PCL polymers underwent thermally-induced unzipping depolymerization process, producing volatile CL monomers. The *c*-PCL polymers did not undergo a statistical rupture of the ester backbone chains as a prelude to the main thermal degradation, which was observed for linear PCL polymers.

Collectively, the TGA analysis confirmed that the thermal stability of PCL can be enhanced significantly by cyclization. This result is quite different from a claim in the literature that the cyclic topology does not have a significant effect on thermal degradation [28].

### 4.3. Phase Transitions (I)

**Figure 6:**
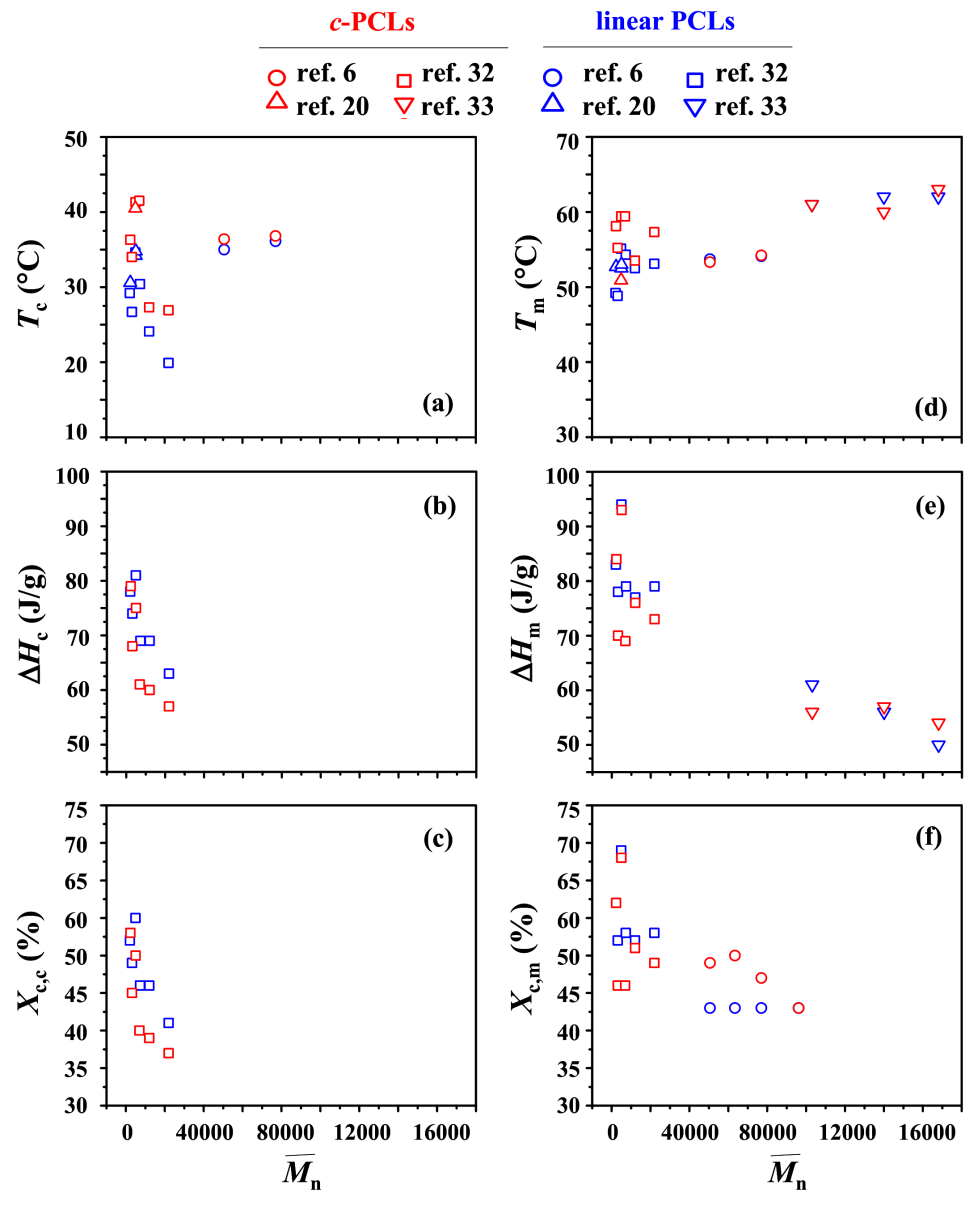
Differential scanning calorimetry (DSC) analysis results of *c*-PCLs and their linear analogues: Here, all data were collected from the literature [6,20,32,33].

The phase transition behaviors of *c*-PCLs and their linear analogues were investigated by several research groups [6,20,31,32]. They were mainly investigated by using differential scanning calorimetry (DSC). All DSC results are plotted together as a function of molecular weight (in particular, $\bar{M_{n}}$) in Figure 6. These plots can provide some features and related questions as follows.

First, *c*-PCL reveals a crystallization exotherm in the DSC analysis. A crystallization temperature *T*_c_ (that corresponds to the temperature at which the exothermic thermogram peak reveals a maximum during cooling with a rate of 10 or 20 °C/min from a melt state) ranges in 27~42 °C depending on the molecular weights (Figure 6a); however, the *T*_c_ values are scattered over that temperature range. In comparison, their linear analogues also show *T*_c_ values scattered over the temperature range 19~37 °C. Overall, it is very hard to find a correlation between *T*_c_ and $\bar{M_{n}}$ over the molecular weight range of 2000 to 168,000 g/mol for *c*-PCLs as well as their linear analogues. These features are quite strange. These unusual results might be caused by several factors such as polydispersity difference, impurity level, incomplete elimination of thermal history, and some differences in the DSC instrumentations and their measurement conditions. However, it is clearly shown that for a given molecular weight *c*-PCL exhibits relatively higher *T*_c_ than that of its linear analogue; their difference still varies with the molecular weight and paired sample set. In particular, the cyclic and linear pairs with $\bar{M_{n}}$ < 30,000 g/mol show relatively large differences in *T*_c_, compared to those of the higher molecular weight pairs. At this moment, this feature could not be understood.

Second, during the cooling process from the melt state, a *c*-PCL of $\bar{M_{n}}$ = 2000 g/mol reveals an exothermic heat of fusion Δ*H*_c_ (78 J/g) which is comparable to that (79 J/g) of its linear analogue; due to these Δ*H*_c_ values, they show almost same crystallinity *X*_c,c_. However, all other *c*-PCLs with $\bar{M_{n}}$ > 2000 g/mol exhibit relatively lower Δ*H*_c_ values, compared to those of their linear counterparts; as a result, the same trend is observed for the *X*_c,c_ values of *c*-PCLs and their linear analogues (Figure 6b,c). Moreover, they reveal a molecular weight dependency whereby both Δ*H*_c_ and *X*_c,c_ decrease as $\bar{M_{n}}$ increases. A similar molecular weight dependency is observed for linear PCLs. Here, at least three questions are raised. Why does the cyclic topology effect take place for the Δ*H*_c_ and *X*_c,c_ of PCL of $\bar{M_{n}}$ > 2000 g/mol? Why is such topology effect not observed for the Δ*H*_c_ and *X*_c,c_ of PCL with $\bar{M_{n}}$ = 2000 g/mol? Why do the Δ*H*_c_ and *X*_c,c_ of *c*-PCL as well as of its linear analogue decrease with increasing molecular weight, respectively?

Third, *c*-PCL shows a crystal melting endotherm in the heating run. A crystal melting temperature *T*_m_ (that corresponds to the temperature at which the endothermic thermogram peak reveals a maximum during heating with a rate of 10 or 20 °C) ranges from 53~63 °C (Figure 6d). In comparison, their linear analogues also show *T*_m_ values over the range of 48~62 °C. These results indicate that *c*-PCL and its linear analogue do not reveal a dependency of *T*_m_ to the molecular weight in a regular manner, respectively. Furthermore, in three pairs *c*-PCLs show relatively lower *T*_m_ values than their linear analogues; on the contrary, for the other pairs *c*-PCLs exhibit relatively higher *T*_m_ values than those of their linear analogues. These collectively suggest that more quantitative studies are still necessary to understand the crystal melting transitions of *c*-PCLs and their linear analogues from the view of the cyclic topology effect.

Fourth, the endothermic heat of fusion Δ*H*_m_ and cystallinity *X*_c,m_ of *c*-PCL measured in a heating run range from 53~63 J/g and 48~68% respectively, depending on the samples and molecular weights (Figure 6e,f). The Δ*H*_m_ and *X*_c,m_ tend to decrease roughly with increasing molecular weight, respectively. In fact, they vary with molecular weight in an irregular manner for the *c*-PCLs with $\bar{M_{n}}$ < 30,000 g/mol. Similar trends of Δ*H*_m_ and *X*_c,m_ are observed for the linear PCLs. These unusual dependencies of Δ*H*_m_ and *X*_c,m_ to the molecular weight could not yet be understood. Furthermore, some *c*-PCLs exhibit lower Δ*H*_m_ and *X*_c,m_ values, compared to those of their linear analogues; the other *c*-PCLs exhibit larger Δ*H*_m_ and *X*_c,m_ values, compared to those of their linear analogues. These results are also very hard to understand from the aspect of the cyclic topology effect.

Fifth, the *X*_c,c_ and *X*_c,m_ values (Figure 6c,f) were calculated from the measured Δ*H*_c_ and Δ*H*_m_ values, respectively, by using linear PCL’s equilibrium heat of fusion $\Delta H_{m}^{o}$ ranged in 135.2~137.3 J/g [6,20,31,32,33,34]. However, higher $\Delta H_{m}^{o}$ values (137~157 J/g) were also reported [43,44,45,46,47,48]. Overall, the $\Delta H_{m}^{o}$ values of linear PCL are scattered over the range 135~157 J/g. Moreover, $\Delta H_{m}^{o}$ still needs to be determined for *c*-PCL.

Sixth, additional research efforts have been made to determine equilibrium melting temperature $T_{m}^{o}$. Linear PCLs were estimated to reveal $T_{m}^{o}$ ranged in 69~98 °C [20,32,35,48,49,50]. In comparison, *c*-PCLs were determined to show $T_{m}^{o}$ ranged in 81~92 °C [20,32,35]. A cyclic topology effect could be discernible on the $T_{m}^{o}$ of PCL but its impact seems not to be significant. More detailed investigation may be necessary.

Lastly, linear PCLs are known to exhibit glass transitions about −60 °C [37,38]. However, the glass transition behavior of *c*-PCL has not yet been investigated in detail.

### 4.4. Phase Transitions (II)

**Figure 7:**
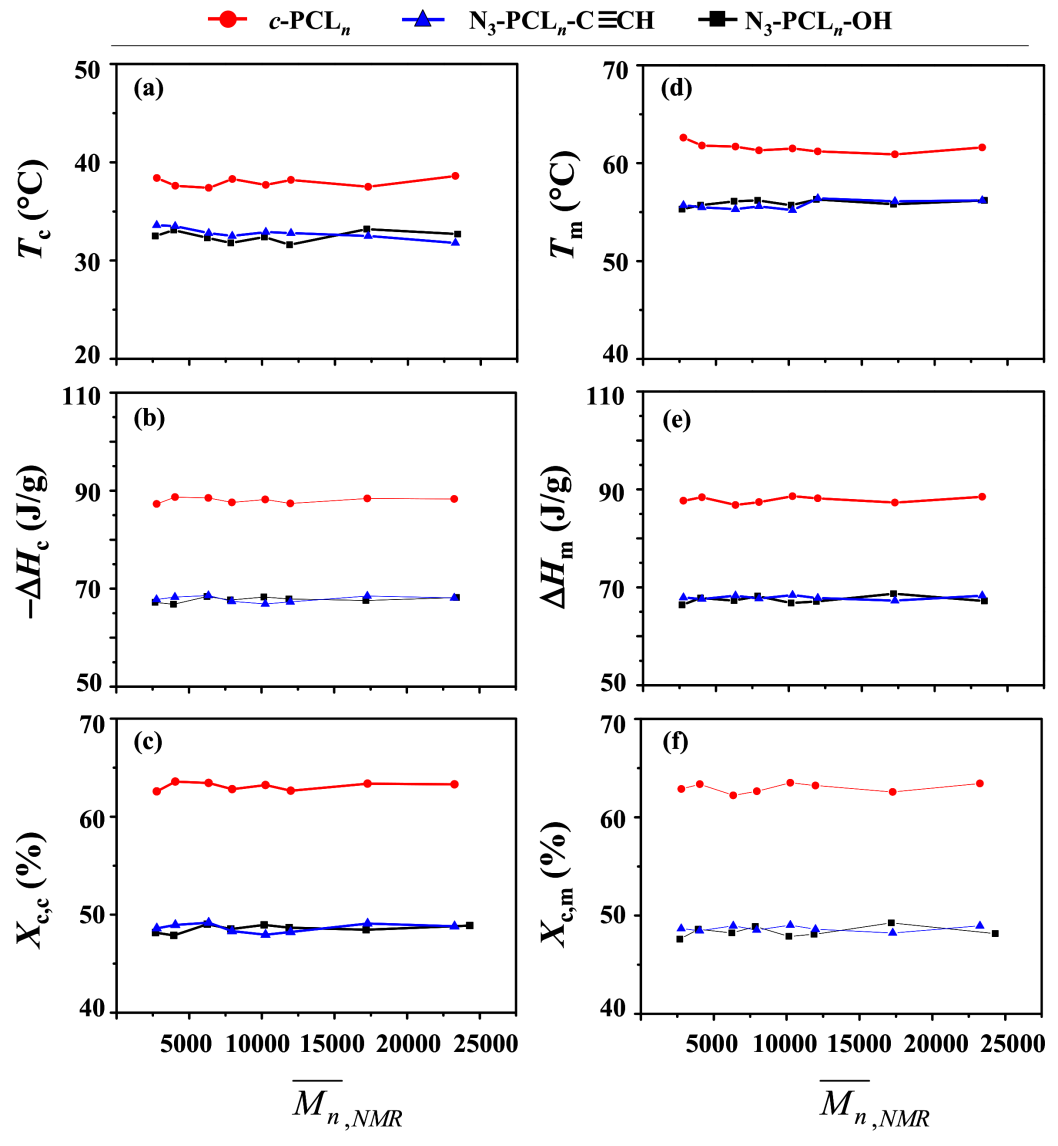
DSC analysis results of *c*-PCLs and their linear analogues synthesized in this study. The measurements were conducted with a rate of 10.0 °C/min under nitrogen atmosphere.

As discussed above, the cyclic topology effect has not been discernible clearly in the thermal transitions of PCL. Furthermore, the thermal transition behaviors of *c*-PCL itself as well as of linear PCL could not be understood in detail. Thus, in this study we investigated them with a series of *c*-PCLs and their linear analogues in a quantitative manner. The DSC analysis results are shown in Table 4 and Figure 7.

In the nonisothermal crystallization with a cooling rate of 10.0 °C/min from the melt state, *c*-PCL reveals a *T*_c_ of 37.4~38.6 °C, a Δ*H*_c_ of −87.3~−88.7 J/g, and a *X*_c,c_ of 62.6~63.6% over the molecular weight range of 2770 to 23,270. These results collectively indicate that the phase transition characteristics of *c*-PCL due to non-isothermal self-assembling show no molecular weight dependencies over the molecular weight range considered. This feature is quite unique and significantly different in part or fully from those reported in the literature [6,20,31,32]. Taking our results into account, the variations in the *T*_c_, Δ*H*_c_ and *X*_c,c_ of *c*-PCL with molecular weight in the literature might indeed originate from substantial amounts of impurities (for examples, unreacted precursors and others) which could not be ignored.

In the case of linear PCLs, no molecular weight dependencies of phase transition characteristics in non-isothermal self-assembling are observed over the molecular weight range 2770 to 23,200 g/mol. These results are quite different in part or fully from those appeared in the literature [6,20,31,32]. Thus, the variations of thermal characteristics reported in the literature may be a strong indication that their linear PCL products could still include certain levels of impurities and have relatively large PDI values.

However, the *c*-PCLs clearly reveal relatively higher *T*_c_ (ca. 5 °C higher), larger Δ*H*_c_ (ca. 10 J/g larger), and larger *X*_c,c_ (14% larger), compared to those of the linear PCL analogues. These results collectively confirm that the cyclic topology effect is quite significant in the crystallization-induced phase transition behaviors of PCL.

In melting at a 10.0 °C/min heating rate of the nonisothermally-crystallized crystals, *c*-PCL exhibits a *T*_m_ of 60.9~62.6 °C, a Δ*H*_m_ of 86.8~88.6 J/g, and a *X*_c,m_ of 62.2~63.5% over the molecular weight range 2770 to 23,200 g/mol. These phase-transition characteristics apparently show no molecular weight dependencies. No molecular weight dependencies are observed on the crystal melting transition characteristics of the linear PCL analogues. However, the *c*-PCL polymers obviously reveal higher *T*_m_ (ca. 6 °C higher), larger Δ*H*_m_ (ca. 10 J/g larger), and larger *X*_c,m_ (14% larger), compared to those of the linear PCL analogues. These comparisons confirm that the cyclic topology effect is also quite significant in the crystal melting transition behaviors of PCL. Overall, this study confirms again that our results are significantly different in part or fully from those reported in the literature [6,20,31,32].

In this study, we have additionally found interesting new features of *c*-PCLs and their linear analogues as follows.

First, *c*-PCL_20_ is an oligomer which has only 2770 g/mol $\bar{M_{n}}$ and 20 $\bar{DP_{n}}$ (number-average degree of polymerization); however it reveals crystallization- and crystal melting-induced phase transition characteristics almost the same with those of higher molecular weight *c*-PCLs. These results suggest that for *c*-PCL’s crystals, the crystallite with a certain thickness (namely, lamellar crystal thickness) is well developed enough with the cyclic chains of $\bar{DP_{n}}$ = 20. The formation of such crystallites seems to be very fast in the low molecular weight polymer as well as in the high molecular weight polymers; thus, the formation kinetics of crystallites could not be distinguishable by varying molecular weight. Furthermore, a surface with a certain level of quality seems to be formed on such crystallites regardless of molecular weights; namely, the surface characteristics of crystallites are not significantly varied with molecular weight.

Second, N_3_-PCL_20_-C≡CH is also an oligomer which has 2770 g/mol $\bar{M_{n}}$ and 20 $\bar{DP_{n}}$, exhibiting crystallization- and crystal melting-induced phase transition characteristics almost same with those of higher molecular weight analogues. These interesting results also suggest that crystallites with a certain thickness are developed enough with the linear chains of only 20 $\bar{DP_{n}}$. Such crystallites may result from a very fast crystallization rate. Such fast crystallization kinetics could not be distinguishable with molecular weights. The crystallite surface characteristics would not easily be differentiated with varying molecular weight either.

Third, all N_3_-PCL*_n_*-OH polymers exhibit almost same phase transition behaviors as the N_3_-PCL*_n_*-C≡CH polymers show. Therefore, very similar crystallite and surface characteristics are expected for the N_3_-PCL*_n_*-OH polymers. Namely, the phase transition characteristics are apparently independent upon the *ω*-hydroxy and ethynyl end groups. These surprising behaviors may be driven by the very fast crystallization rate of PCL main body; namely, the effect of chain end groups cannot be easily discernible under such fast crystallizations.

Fourth, Δ*H*_c_ ≅ Δ*H*_m_ for a given *c*-PCL as well as for a chosen linear PCL. These results are a good evidence that the crystallization rate is very fast for *c*-PCLs as well as for their linear analogues.

Fifth, such fast crystallization characteristics of linear PCLs as well as of cyclic analogues may originate from a highly optimized chain mobility (which is essential for self-assembling) due to their short persistent length or Kuhn length (namely, high chain flexibility; its information is available in the literature [38]) and favorable interchain and intrachain interactions using the ester linkers in the repeat units.

Sixth, here one question arises: why does *c*-PCL always reveal higher *T*_c_ (ca. 5 °C higher) and larger Δ*H*_c_ (ca. 10 J/g larger) than those of its linear analogues in non-isothermal crystallization from the melt? For these phenomena, there are several factors to consider: (i) degree of supercooling Δ*T*_c_ (due to $T_{m}^{o}$); (ii) chain mobility; (iii) degree of entanglement; (iv) degree of molecular preordering; and (v) presence of chain-ends or chain-endless. In general, higher $T_{m}^{o}$ in a polymer can induce higher *T*_c_, leading to larger Δ*T*_c_ in crystallization. Taking this fact into account, *c*-PCL may own relatively higher $T_{m}^{o}$ than those of its linear analogues; higher $T_{m}^{o}$ (2 to 11 °C higher) was reported for *c*-PCL [20,33] but still controversial [48,49,50]. The crystallites with lower Δ*H*_c_ are generally formed under a crystallization condition with higher Δ*T*_c_. However, *c*-PCLs reveal larger Δ*H*_c_, compared to those of their linear analogues. The observation of such larger Δ*H*_c_ suggests that *c*-PCLs underwent much faster crystallizations, compared to those of their linear analogues; relatively faster crystallization rates were reported for *c*-PCLs [6,20,33,42]. *c*-PCLs were reported to reveal higher chain mobility (i.e., faster diffusion) in the melt state, compared to their linear analogues because of their compact size due to the cyclic chain system without chain ends. This factor may make a positive contribution to the larger Δ*H*_c_ values of *c*-PCLs. In fact, it is well known that two ends in a linear polymer can accelerate the chain mobility by their high entropic gains. However, it seems that the impact of two chain ends on the chain mobility of linear PCL is relatively lower, compared to that of the compact size without chain ends on the chain mobility of its cyclic analogue. The faster diffusions of *c*-PCLs could be attributed to less degree of entanglement (or disentanglement) in addition to the compact geometrical dimension. In particular, all *c*-PCLs of our study were synthesized in a very dilute condition so that they are very much disentangled or less entangled. *c*-PCLs might be in a preordered state rather than completely random coil like state because the ester linkers in the repeat units can induce a certain level of attractive interactions intra- and inter-molecularly. The presence of such preordered polymer chains can make a positive contribution to the faster crystallization and the higher ordering in the resulting crystallites. This effect is more favorable for *c*-PCLs rather than their linear analogues owning two chain-ends which can have significant negative impacts on such preordering of chains. All factors considered here originate in the absence of chain-ends in *c*-PCLs; namely, the cyclic topological effect is significant in PCL.

Lastly, another question arises: why does *c*-PCL always exhibit higher *T*_m_ (ca. 6 °C higher) with larger Δ*H*_m_ (ca. 10 J/g larger) than those of its linear analogues in the crystal melting process? In general, higher quality crystals in a polymer reveal higher *T*_m_ and larger Δ*H*_m_. Therefore, the higher *T*_m_ and larger Δ*H*_m_ of *c*-PCLs may be attributed to their crystals possibly associated with (i) larger crystallite thickness; (ii) higher lateral ordering; (iii) less defect level in the crystallites and their surfaces; and (iv) a positive contribution of entropy term which originates from the free chain-ends and cyclic topology, compared to those of their linear analogues. However, these speculations still need to be confirmed experimentally. Moreover, to rank these three factors in the aspect of contributions, more information is necessary.

## 5. Crystallization Characteristics

Córdova et al. [35] and Pérez [6] reported isothermal crystallizations of *c*-PCLs with ${\bar{M_{n}}}_{,MALDI-TOFF}$ = 2000~22,000 g/mol (which were prepared by azide–alkyne click cyclizations) and their linear analogues with *α*-azido and *ω*-hydoxyl or *ω*-acetyl end groups (which were synthesized with the aid of tin octanoate catalyst) over the temperature range of 38–56 °C by using DSC and polarized light optical microscopy (PLOM). They found that regardless of the chain topologies, all oligomeric PCLs undergo crystallization, forming spherulites instantaneously (Avrami exponent *n* = 3) or sporadically (*n* = 4). For the linear analogues, the crystallization behaviors were not influenced by the difference between the *ω*-end groups. However, *c*-PCLs always showed faster crystallization rates, compared to those of the linear counterparts. The PLOM analysis further found that the nucleation density at saturation values (long times) was 7% higher for *c*-PCL (4900 g/mol ${\bar{M_{n}}}_{,MALDI-TOFF}$) than for the linear counterpart at the same *T*_c_ (54 °C); the nucleation rate was much higher for *c*-PCL, which was a clear sign of a more instantaneous nucleation process in *c*-PCL as compared to the linear counterpart. Overall, the nucleation and spherulite growth analysis with PLOM, as well as the DSC data analysis with the Lauritzen–Hoffman theory confirmed that such faster overall crystallization rates of *c*-PCLs are mainly driven by the crystal growth rates and attributed in part to the nucleation rates. $T_{m}^{o}$ was determined to be 81 °C for *c*-PCLs and 80 °C for the linear analogues by Hoffman–Week analysis. Therefore, they interpreted the faster crystallization rates by considering higher chain diffusions (i.e., mobilities) of *c*-PCLs in more collapsed conformation (which are due to the cyclic topology in free of chain ends) rather than the differences in the degrees of supercooling (Δ*T*) caused by the $T_{m}^{o}$ values associated with the chain topologies.

The above PCL samples were reexamined by Su et al. [32]. They measured $T_{m}^{o}$ = 91.2 °C for *c*-PCLs (which is much higher than that (81 °C) reported previously [35]) and 80.0 °C for the linear analogues by using the Thomson–Gibbs analysis. Namely, they found a large difference (11.2 °C) between the $T_{m}^{o}$ values of *c*-PCLs and the linear counterparts. Thus, they claimed that the faster crystallization rates of *c*-PCLs at given temperatures could be attributed to the significantly larger degree of supercooling. Moreover, Wang et al. [8] and Li et al. [7] measured slower crystallization rates of *c*-PCLs (2000 and 4900 g/mol ${\bar{M_{n}}}_{,MALDI-TOFF}$) in the low temperature region, compared to those of the linear counterpart. They claimed that such the result could be attributed to a higher mobility of free chain ends in the linear analogue as compared to the *c*-PCL. Such faster crystallization rates of linear PCLs could not be observed for a linear PCL with ${\bar{M_{n}}}_{,MALDI-TOFF}$ = 22,000 g/mol because of diluted chain-end effects in long chain polymers.

NMR and DSC analyses were conducted on the crystallizations of *c*-PCLs with ${\bar{M_{n}}}_{,GPC}$ = 50,000~80,000 g/mol and 1.6~2.1 PDI (which were prepared by ring-expansion polymerization with a cyclic tin catalyst and indeed contained a tin-linker in the ring) and their linear analogues [31]. The analyses confirmed relatively higher crystallinities for the isothermally-crystallized *c*-PCLs, compared to those of the linear PCL analogues. This study claimed that such higher crystallinities were attributed to a higher chain segmental mobility of the *c*-PCLs, which was measured in the melt state by using NMR and rheology analyses. The enhanced overall segmental mobility in the melt could lead to more perfect morphology, resulting in higher crystallinity. The crystallization analysis with the aid of nucleating agents further found that *c*-PCLs underwent faster crystal growth than that of the linear analogues.

Together with X-ray scattering, DSC analysis was extended to crystallization behaviors of *c*-PCLs with 75,000~142,000 g/mol ${\bar{M_{w}}}_{,light\;scattering}$ (weight-average molecular weight measured by light scattering) and 1.83~2.03 PDI (which were prepared by Zwitterionic polymerization and cyclization and thus in free of heterogeneous linkers such as tin and triazole) and their linear counterparts [20]. This study determined $T_{m}^{o}$ = 84.2 °C for *c*-PCL and 82.0 °C for linear PCL by using Hoffman–Weeks analysis but $T_{m}^{o}$ = 121 °C for *c*-PCL and 97 °C for linear PCL by the Thomson–Gibbs analysis. The study confirmed that all *c*-PCLs crystallized more rapidly than the linear analogues. The X-ray scattering analysis found that *c*-PCLs as well as the linear counterparts reveal increments in the crystal thickness as the crystallization temperature increases. Overall, this study concluded that the cyclic topology can have a significant influence on the rate of crystallization from the melt but does not have a significant influence on the crystal structure or morphology.

As reviewed above, all studies found that at a given crystallization temperature from the melt, *c*-PCLs always exhibited faster crystallization rates than those of the linear counterparts although investigated in a limited base and further in free of or with heterogeneous linkers in the ring structure. However, their interpretations and understandings on such crystallization behaviors were controversial. Regarding the degree of supercooling in crystallization, $T_{m}^{o}$ values determined for *c*-PCL as well as for linear PCL were also controversial. Therefore, detailed investigation is still needed to understand the crystallization behaviors of *c*-PCL in depth.

## 6. Morphological Characteristics

By contrast with the synthesis and properties of *c*-PCLs, their morphological characteristics have rarely been investigated. Only two reports are available in the literature as reviewed below.

### 6.1. Transmission Electron Microscopy (TEM), Electron Diffraction and Atomic Force Microscopy (AFM) Analyses

Transmission electron microscopy (TEM) and electron diffraction analyses, as well as atomic force microscopy (AFM) analysis were conducted for the lamellar crystals of a *c*-PCL (which was prepared by azide–alkyne click cyclization; ${\bar{M_{n}}}_{,MALDI-TOFF}$ = 2320~7000 g/mol and PDI = 1.08~1.13) and its linear analogues with *α*-azido and *ω*-hydoxyl or *ω*-acetyl end groups (which were synthesized with the aid of tin octanoate catalyst; ${\bar{M_{n}}}_{,MALDI-TOFF}$ = 2040~7340 g/mol and PDI = 1.11~1.17) grown successfully in *n*-hexanol and *N*,*N*-dimethylacetamide (DMAc) solutions [32]. The TEM analysis found that both the *c*-PCL and its linear counterparts made distorted hexagonal-shaped lamellar crystals in a size of 2~10 μm through crystallizations in the dilute solutions. The solution-grown *c*-PCL crystal revealed a typical single crystallographic image in electron diffraction, which was identical to those of the linear counterpart crystals; more than 25 reflection spots were clearly discernible and indexed with an orthorhombic lattice (*a* = 0.747 nm, *b* = 0.498 nm and *c* = 1.705~1.729 nm) with a space group of *P*2_1_2_1_2_1_. These single crystals exhibited relatively higher *T*_m_ values (3~4 °C higher), compared to those of the melt-crystallized ones. The AFM analysis found that the interlamellar distance *L* (i.e., long period) ranged from 5.4~13.2 nm for the *c*-PCL crystals and 8.7~11.6 nm for the linear PCL crystals (here, *α*-azido-*ω*-hydoxyl-PCL crystals). Both *c*-PCL and linear PCL crystals showed a trend that the *L* value increases with increasing molecular weight. The *c*-PCL of ${\bar{M_{n}}}_{,MALDI-TOFF}$ = 2320 g/mol made relatively thinner lamellar crystals (*L* = 5.4 nm) than those (8.7 nm) of the linear counterpart. In contrast, the *c*-PCLs of >2320 g/mol ${\bar{M_{n}}}_{,MALDI-TOFF}$ formed thicker lamellar crystals, compared to those of the linear counterparts. These results collectively suggest that the cyclic topology effect is present in the solution-grown morphology of PCL and, however, its impact is varied negatively or positively depending on the molecular weight; these statements are standing on the very limited observations. Thus, more detailed investigation is still needed to understand the cyclic topology effect on the solution-grown lamellar structure of PCL.

### 6.2. Transmission Small-Angle X-ray Scattering (TSAXS) Analysis

Shin et al. [20] reported time-resolved transmission small-angle X-ray scattering (TSAXS) analysis on the crystallizations of *c*-PCLs (${\bar{M_{w}}}_{,light\;scattering}$ = 75,000~142,000 g/mol and PDI = 1.83~2.03; all were prepared by Zwitterionic polymerization and cyclization) and their linear counterparts. They attempted to analyze the measured TSAXS data by adopting a correlation function approach with two-phase model. The TSAXS analysis confirmed that *c*-PCLs formed lamellar structures via crystallizations from the melt as linear PCLs demonstrated. During the isothermal crystallization of *c*-PCL at a chosen temperature *T*_c_, the long period *L* (, i.e., interlamellar distance) was decreased as the crystallization time increased; but the crystal thickness *l*_c_ apparently was not varied with the crystallization time. The *l*_c_ value was slightly increased with *T*_c_ in an irregular manner; namely, the *l*_c_ values scattered with varying *T*_c_. This *T*_c_ dependency of *l*_c_ may directly correlate to the unusually high $T_{m}^{o}$ (121 °C) of *c*-PCL estimated by Thomson–Gibbs analysis. Similar morphological characteristics were observed for the linear PCLs. However, the *l*_c_ value was increased with *T*_c_ in a regular manner than an irregular manner. For the specimens crystallized isothermally at a chosen *T*_c_, *c*-PCL revealed an *L* value and an *l*_c_ value, which were very similar to those of the linear PCL. For example, both *c*-PCLs and their linear counterparts, which were crystallized at 45 °C, showed *L* = ca. 20 nm and *l*_c_ = ca. 9 nm. Overall this study concluded that the lamellar structure characteristics of PCL were insensitive to the cyclic topology.

Su et al. [32] extended TSAXS analysis to a *c*-PCL (${\bar{M_{n}}}_{,MALDI-TOFF}$ = 7000 g/mol and PDI = 1.11) and its linear analogue (*α*-azido-*ω*-acetyl-PCL; ${\bar{M_{n}}}_{,MALDI-TOFF}$ = 7340 g/mol and PDI = 1.13) crystallized isothermally at various temperatures. *c*-PCL was determined to show a trend that the *L* value was almost linearly increased from 12.4 to 14.9 nm as *T*_c_ was increased from 20 to 55 °C. In case of the linear PCL, the *L* was also increased with increasing *T*_c_ but varied in an irregular manner; the *L* values ranged in 13.5~15.0 nm over *T*_c_ = 25~53 °C. The *l*_c_ value was further estimated in a qualitative manner rather than a quantitative way. For *c*-PCL, the *l*_c_ value was linearly increased from 7.25 to 8.75 nm as *T*_c_ was increased from 20 to 55 °C. For the linear PCL, the *l*_c_ value was also linearly increased from 7.30 to 8.40 nm over *T*_c_ = 25~53 °C. Considering the irregular variations of *L* with *T*_c_, such linear increments in the *l*_c_ variation with *T*_c_ are quite surprising. Overall, *c*-PCL revealed relatively smaller *L* values than those of the linear PCL; but, *c*-PCL exhibited slightly larger *l*_c_ values than those of the linear PCL. These results collectively indicate that the morphological characteristics of PCL could be influenced by the cyclic topology.

As reviewed above, only two X-ray scattering studies have been reported for *c*-PCLs so far. Unfortunately, their results are controversial in the aspect of cyclic topology effect. Therefore, more detailed X-ray scattering analysis is still necessary to get better features of the cyclic topology effect in the morphological structure of PCL.

## 7. Summary and Perspectives

Several synthetic schemes have been successfully developed to produce *c*-PCLs, as a result of rigorous research effort: (a) two ring-expansion polymerization schemes; (b) seven intramolecular cyclization schemes; (c) one intermolecular cyclization scheme. In addition, it was demonstrated that a comprehensive, highly-efficient purification process, which combines several essential workup methods including chromatographic separation and purification, is essential for obtaining high-purity *c*-PCL. Nevertheless, several issues remain in the synthesis of *c*-PCL as follows. First, all developed synthetic methods still find it challenging to minimize or completely eliminate byproducts. Second, the synthetic schemes using metal-containing initiators and/or catalysts need to be further developed to produce *c*-PCL products free of metal. Third, the cyclization schemes need to further improve reaction yields. Lastly, it is a challenge to develop a more efficient workup process for producing high-purity *c*-PCL products in large quantity.

The chain structure characteristics and properties of *c*-PCL have been examined on a limited basis: hydrodynamic volume, solution and melt viscosities, and chain mobility in the melt, hydrolysis, thermal degradation, crystallization, nucleation, spherulitic growth, crystal melting, equilibrium melting temperature, and equilibrium heat of fusion. The effect of cyclic topology was evidenced in the chain characteristics (hydrodynamic volume, solution and melt viscosities, and chain mobility in the melt) as well as in some of the properties. However, for the other properties the cyclic topology effects were controversial. The morphological structures of *c*-PCLs and their linear counterparts have been investigated on a very limited basis. Nevertheless, the structural results were also controversial in the aspect of the cyclic topology effect. Such controversial results may be attributed in part to the impurities and their quantities in *c*-PCL products, which cannot be neglected. Therefore, more quantitative and comprehensive investigations are urgently required for producing high-purity *c*-PCLs and their linear counterparts in order to gain a better understanding of them and to develop appropriate applications.

## Figures and Tables

**Table 1:** Ring opening polymerization of *ε*-caprolactone (CL) using 6-azido-1-hexanol initiator and diphenyl phosphate catalyst in toluene *^a^*.

Polymer	[M]/[I] *^b^*	Reaction Time (h)	Yield *^c^* (%)	*M_n_*_,*theo*_ *^d^* (g/mol)	${\bar{M_{n,}}}_{NMR}$ *^e^* (g/mol)	${\bar{M_{n,}}}_{GPC}$ *^f^* (g/mol)	PDI *^g^*
N_3_-PCL_20_-OH	20	2.0	96.5	2440	2680	2700	1.10
N_3_-PCL_30_-OH	30	5.0	97.3	3600	3940	4900	1.06
N_3_-PCL_50_-OH	50	8.0	97.2	5900	6250	7700	1.09
N_3_-PCL_70_-OH	70	10.0	96.5	8200	7850	10,700	1.09
N_3_-PCL_90_-OH	90	14.0	96.8	10,500	10,100	12,600	1.08
N_3_-PCL_100_-OH	100	20.0	96.4	11,700	11,900	16,200	1.06
N_3_-PCL_150_-OH	150	29.0	97.3	17,400	17,200	20,800	1.07
N_3_-PCL_200_-OH	200	42.0	97.1	23,100	24,300	26,400	1.07

*^a^* Reaction temperature, 25 °C; [M], 1.0 mol/L. *^b^* Molar ratio of initial monomer and initiator. *^c^* Polymerization yield. *^d^* Molecular weight calculated from the conversion and the monomer to initiator ratio: [([M]/[I]) × conversion × (*M_w_* of CL)] + (*M_w_* of I). *^e^* Number-average molecular weight determined by ^1^H nuclear magnetic resonance (NMR) spectroscopy analysis. *^f^* Number-average molecular weight determined THF by using gel permeation chromatography (GPC) analysis calibrated with polystyrene (PS) standards. *^g^* Polydispersity index determined by using GPC analysis calibrated with PS standards.

**Table 2:** Synthesis results of N_3_-PCL*_n_*-C≡CH precursors and their *c*-PCL*_n_* products.

Polymer	Yield *^a^* (%)	*M_n_*_,*theo*_ *^b^* (g/mol)	${\bar{M_{n,}}}_{NMR}$ *^c^* (g/mol)	${\bar{M_{n,}}}_{GPC}$ *^d^* (g/mol)	PDI *^e^*
N_3_-PCL_20_-C≡CH	96.8	2530	2770	2700	1.10
*c*-PCL_20_	37.7	2540	2790	2300	1.07
N_3_-PCL_30_-C≡CH	97.7	3690	4000	5000	1.09
*c*-PCL_30_	38.1	3700	4100	3700	1.09
N_3_-PCL_50_-C≡CH	97.2	5990	6340	7700	1.06
*c*-PCL_50_	37.9	6000	6350	5200	1.07
N_3_-PCL_70_-C≡CH	97.5	8300	7950	11,100	1.08
*c*-PCL_70_	39.5	8300	8000	7900	1.10
N_3_-PCL_90_-C≡CH	98.1	10,600	10,200	13,000	1.10
*c*-PCL_90_	38.9	10,700	10,200	9500	1.08
N_3_-PCL_100_-C≡CH	96.8	11,700	11,900	16,400	1.10
*c*-PCL_100_	40.2	11,800	12,000	12,100	1.06
N_3_-PCL_150_-C≡CH	97.5	17,500	17,200	20,800	1.11
*c*-PCL_150_	40.5	17,500	17,300	15,300	1.13
N_3_-PCL_200_-C≡CH	97.6	23,200	23,200	26,800	1.13
*c*-PCL_200_	41.1	23,300	23,200	21,500	1.11

*^a^* Reaction yield. *^b^* Molecular weight estimated from the theoretical molecular weights of N_3_-PCL*_n_*-OH precursors in Table 1. *^c^* Number-average molecular weight determined by ^1^H NMR spectroscopy analysis. *^d^* Number-average molecular weight determined THF by using GPC analysis calibrated with polystyrene (PS) standards. *^e^* Polydispersity index determined by using GPC analysis calibrated with PS standards.

**Table 3:** Preparative GPC analysis results of the reaction mixtures obtained by click cyclization reactions of N_3_-PCL*_n_*-C≡CH precursors.

Precursor Reactant	Reaction Mixture *^a^*		
	*c*-PCL *^b^* (%)	Cyclic Byproducts *^c^* (%)	Linear Polymers *^d^* (%)
N_3_-PCL_20_-C≡CH	37.7	8.7	53.6
N_3_-PCL_30_-C≡CH	38.1	3.8	58.1
N_3_-PCL_50_-C≡CH	37.9	6.3	55.8
N_3_-PCL_70_-C≡CH	39.5	8.3	52.2
N_3_-PCL_90_-C≡CH	38.9	0.2	60.9
N_3_-PCL_100_-C≡CH	40.2	4.0	55.8
N_3_-PCL_150_-C≡CH	40.5	2.6	56.9
N_3_-PCL_200_-C≡CH	41.1	0.4	58.5

*^a^* Reaction mixture obtained by click cyclization reaction. *^b^* Cyclic PCL obtained by the separation and fractionation using a preparative GPC system after treated with PS-C≡CH resins. *^c^* Cyclic byproducts obtained by the separation and fractionation using a preparative GPC system after treated with PS-C≡CH resins. *^d^* Sum of the unreacted precursor polymers and possible linear byproducts removed by treatment with PS-C≡CH resins; this was estimated from the yield of cyclic PCL product and the preparative GPC analysis result.

**Table 4:** Thermal transition characteristics of *c*-PCL*_n_* products and their linear analogues.

Polymer *^a^*	${\bar{M_{n,}}}_{NMR}$ *^b^* (g/mol)	PDI *^c^*	*T*_c_ *^d^* (°C)	Δ*H*_c_ *^e^* (J/g)	*X*_c,m_ *^f^* (%)	*T*_m_ *^g^* (°C)	Δ*H*_m_ *^h^* (J/g)	*X*_c,m_ *^i^* (%)
*c*-PCL_20_	2790	1.07	38.4	−87.3	62.6	62.6	87.7	62.9
N_3_-PCL_20_-C≡CH	2770	1.10	33.6	−67.8	48.6	55.7	67.9	48.7
N_3_-PCL_20_-OH	2680	1.10	32.5	−67.2	48.2	55.3	66.4	47.6
*c*-PCL_30_	4100	1.09	37.6	−88.7	63.6	61.8	88.4	63.4
N_3_-PCL_30_-C≡CH	4000	1.09	33.5	−68.3	49.0	55.5	67.6	48.5
N_3_-PCL_30_-OH	3940	1.06	33.1	−66.8	47.9	55.7	67.8	48.6
*c*-PCL_50_	6350	1.07	37.4	−88.5	63.4	61.7	86.8	62.2
N_3_-PCL_50_-C≡CH	6340	1.06	32.8	−68.6	49.2	55.3	68.3	49.0
N_3_-PCL_50_-OH	6250	1.09	32.3	−68.4	49.0	56.1	67.3	48.2
*c*-PCL_70_	8000	1.10	38.3	−87.6	62.8	61.3	87.4	62.7
N_3_-PCL_70_-C≡CH	7950	1.08	32.5	−67.4	48.3	55.6	67.7	48.5
N_3_-PCL_70_-OH	7850	1.09	31.8	−67.7	48.5	56.2	68.2	48.9
*c*-PCL_90_	10,200	1.08	37.7	−88.2	63.2	61.5	88.6	63.5
N_3_-PCL_90_-C≡CH	10,200	1.10	32.9	−66.9	48.0	55.2	68.4	49.0
N_3_-PCL_90_-OH	10,100	1.08	32.4	−68.3	49.0	55.7	66.8	47.9
*c*-PCL_100_	12,000	1.06	38.2	−87.4	62.7	61.2	88.2	63.2
N_3_-PCL_100_-C≡CH	11,900	1.10	32.8	−67.3	48.2	56.4	67.8	48.6
N_3_-PCL_100_-OH	11,900	1.06	31.6	−67.9	48.7	56.3	67.1	48.1
*c*-PCL_150_	17,300	1.13	37.5	−88.4	63.4	60.9	87.3	62.6
N_3_-PCL_150_-C≡CH	17,200	1.11	32.5	−68.5	49.1	56.1	67.3	48.2
N_3_-PCL_150_-OH	17,200	1.07	33.2	−67.6	48.5	55.8	68.7	49.3
*c*-PCL_200_	23,200	1.11	38.6	−88.3	63.3	61.6	88.5	63.4
N_3_-PCL_200_-C≡CH	23,200	1.13	31.8	−68.1	48.8	56.2	68.3	49.0
N_3_-PCL_200_-OH	24,300	1.07	32.7	−68.2	48.9	56.2	67.2	48.2

*^a^* Polymers in Table 1 and Table 2. *^b^* Number-average molecular weight determined by ^1^H NMR spectroscopy analysis (Table 1 and Table 2). *^c^* Polydisperty index determined by using GPC analysis calibrated with PS standards (Table 1 and Table 2). *^d^* Crystallization temperature at the peak maximum of the crystallization transition in DSC analysis with a cooling rate of 10.0 °C/min. *^e^* Heat of fusion for crystallization in cooling run from melt with a rate of 10.0 °C/min. *^f^* Crystallinity estimated from the heat of fusion of crystallization by assuming $\Delta H_{c}^{o}$ = −139.5 J/g. *^g^* Temperature at the peak maximum of the crystal melting transition in DSC analysis with a heating rate of 10.0 °C/min. *^h^* Heat of fusion for crystal melting in heating run with a rate of 10.0 °C/min. *^i^* Crystallinity estimated from the heat of fusion of crystal melting by assuming $\Delta H_{m}^{o}$ = 139.5 J/g [51].
